# RNA sequencing reveals resistance of TLR4 ligand-activated microglial cells to inflammation mediated by the selective jumonji H3K27 demethylase inhibitor

**DOI:** 10.1038/s41598-017-06914-5

**Published:** 2017-07-26

**Authors:** Amitabh Das, Sarder Arifuzzaman, Taeho Yoon, Sun Hwa Kim, Jin Choul Chai, Young Seek Lee, Kyoung Hwa Jung, Young Gyu Chai

**Affiliations:** 1 Institute of Natural Science & Technology, Hanyang University, Ansan, 15588 Republic of Korea; 2 Department of Bionanotechnology, Hanyang University, Seoul, 04673 Republic of Korea; 3 Department of Molecular & Life Sciences, Hanyang University, Ansan, 15588 Republic of Korea

## Abstract

Persistent microglial activation is associated with the production and secretion of various pro-inflammatory genes, cytokines and chemokines, which may initiate or amplify neurodegenerative diseases. A novel synthetic histone 3 lysine 27 (H3K27) demethylase JMJD3 inhibitor, GSK-J4, was proven to exert immunosuppressive activities in macrophages. However, a genome-wide search for GSK-J4 molecular targets has not been undertaken in microglia. To study the immuno-modulatory effects of GSK-J4 at the transcriptomic level, triplicate RNA sequencing and quantitative real-time PCR analyses were performed with resting, GSK-J4-, LPS- and LPS + GSK-J4-challenged primary microglial (PM) and BV-2 microglial cells. Among the annotated genes, the transcriptional sequencing of microglia that were treated with GSK-J4 revealed a selective effect on LPS-induced gene expression, in which the induction of cytokines/chemokines, interferon-stimulated genes, and prominent transcription factors TFs, as well as previously unidentified genes that are important in inflammation was suppressed. Furthermore, we showed that GSK-J4 controls are important inflammatory gene targets by modulating STAT1, IRF7, and H3K27me3 levels at their promoter sites. These unprecedented results demonstrate that the histone demethylase inhibitor GSK-J4 could have therapeutic applications for neuroinflammatory diseases.

## Introduction

Microglial cells are the resident macrophages of the brain and spinal cord and act as the first-line active immune defense as well as in brain-specific innate immune responses in the central nervous system (CNS). Through the interaction of various infectious agents, including bacterial pathogens, microglial cells become activated and react quickly^1^. The priming of microglia is associated with the production and release of numerous pro-inflammatory mediators, including reactive oxygen species (ROS), nitric oxide (NO), prostaglandins (PGs), cytokines and chemokines. Prolonged or excessive microglial activation may promote pathological forms of inflammation that contribute to the initiation and progression of neurodegenerative diseases, including Alzheimer’s disease (AD), Parkinson’s disease (PD) and multiple sclerosis (MS)^2, 3^. However, the defensive or preventive mechanisms against the detrimental phenotypes associated with microglial cells have not been fully understood. Considering the significant impact of microglial cells in innate immune functions, preventing their activation may be important in the search for neurodegenerative diseases treatment options.

Morphologically, the microglial cell surface is furnished with various pattern recognition receptors (PRRs), including the Toll-like receptor (TLR) family (denoted as TLR1-9), to detect and respond to the presence of various stimuli and toxins^4, 5^. The bacterial endotoxin lipopolysaccharide (LPS), which is mostly recognized by TLR4, activates intracellular signaling pathways and increases the expression of pro-inflammatory mediators such as NO, prostaglandin E2 (PGE2), cyclooxygenase-2 (COX-2), cytokines (e.g., IL1B, IL6 and TNF-α) and TFs (e.g., NF-κB, IRF, STAT) in microglial cells^6^. From previous studies, the response of macrophages to LPS is believed to proceed through histone modification at specific inflammatory genes, prompting further exploration to address the temporal cascade of epigenetic events and the effects of specific epigenetic inhibitors. A pivotal study showed that upon LPS stimulation, the H3K27 demethylase JMJD3 was rapidly induced and that more than 70% of LPS-induced genes recruited JMJD3 to their promoter region, which is a general hallmark of gene activation^7^. Indeed, JMJD3 is a bona fide mediator of H3K27me1/me2/me3 demethylation, by reprogramming the transcription of genes by recruiting distinct TFs to gene promoters via epigenetic regulation that is involved in pro-inflammatory gene transcription^7–9^. These observations raised the possibility that JMJD3 may contribute to demethylation-dependent histone-packaged inflammatory gene expression programs associated with various human diseases.

Recently, a potent and highly specific JMJD3 inhibitor, GSK-J4, was discovered by Kruidenier and colleagues^10^. GSK-J4 reduces pro-inflammatory cytokine production by modulating JMJD3, leading to a reduction in H3K27me3 in LPS-induced macrophage cells^11, 12^. Recently, GSK-J4 has been demonstrated to exhibit a potent inhibitory activity against a range of cell lines derived from certain cancers, including T-ALL, B-ALL and glioma^11, 12^. These studies empower the mechanistic investigation of how this inhibitor can be used to effectively modulate JMJD3 in microglial cells.

Although GSK-J4 decreases the production of inflammatory cytokines in LPS-induced macrophages^10^, a genome-wide search for GSK-J4 molecular targets in LPS-induced microglial cells has not yet been performed. Therefore, we studied gene array and comparative gene expression analyses from PM and BV-2 microglial cells upon stimulation with LPS, GSK-J4, and LPS + GSK-J4 using massively parallel cDNA sequencing (RNA-seq), which opened the way to unbiased and efficient assays on the transcriptome of any mammalian cell^13, 14^. In principle, RNA-seq allows the identification of all expressed transcripts, both protein-coding and non-coding. Furthermore, several studies indicate that next-generation sequencing is more valuable and particularly appropriate to examine the pathogenesis of complex neurodegenerative diseases such as AD^15^. To the best of our knowledge, this is the first genome-wide study of GSK-J4-mediated global gene expression changes in PM and BV-2 microglial cells using RNA-seq analysis.

Here, for the first time, we report that GSK-J4 is a potent modulator of microglial activation. In particular, GSK-J4 treatment resulted in the significant down-regulation of key inflammatory genes in LPS-induced PM and BV-2 microglial cells. Importantly, these inflammatory genes were not affected by GSK-J4 treatment alone. Overall, the results suggested that the synthetic compound GSK-J4 might be an effective therapeutic target with possible research and clinical value. Taken together, these findings establish a role for GSK-J4 in mouse microglia stimulation and justify the further testing of GSK-J4- targeting genes in the treatment of inflammatory brain damage.

## Results

### Distinct gene signatures were identified during the inflammatory response using RNA-seq analysis in PM and BV2 microglial cells

To determine the high-resolution transcriptome in response to LPS stimulation, we treated PM and BV-2 microglial cells with LPS for 4-h before cDNA library preparation for RNA-seq experiments. The RNA-seq transcriptional analysis was performed using three independent samples (biological replicates) of each treatment in PM. The data from all the experiments (each group) were combined, and the genes whose expression levels significantly differed were identified. We used a 1% false discovery rate (FDR), *P* ≤ 0.01, and log_2_-fold change ≥1.5 for up- or down-regulation as the criteria for defining the differentially expressed genes. Of note, we previously found that most of the inflammatory response-related genes were up-regulated at a 4-h time point^16, 17^. We chose this time point for transcriptional profiling; this time point was also used in other studies^18, 19^ that investigated the general induction pattern of microglial activation by LPS.

**Figure 1:**
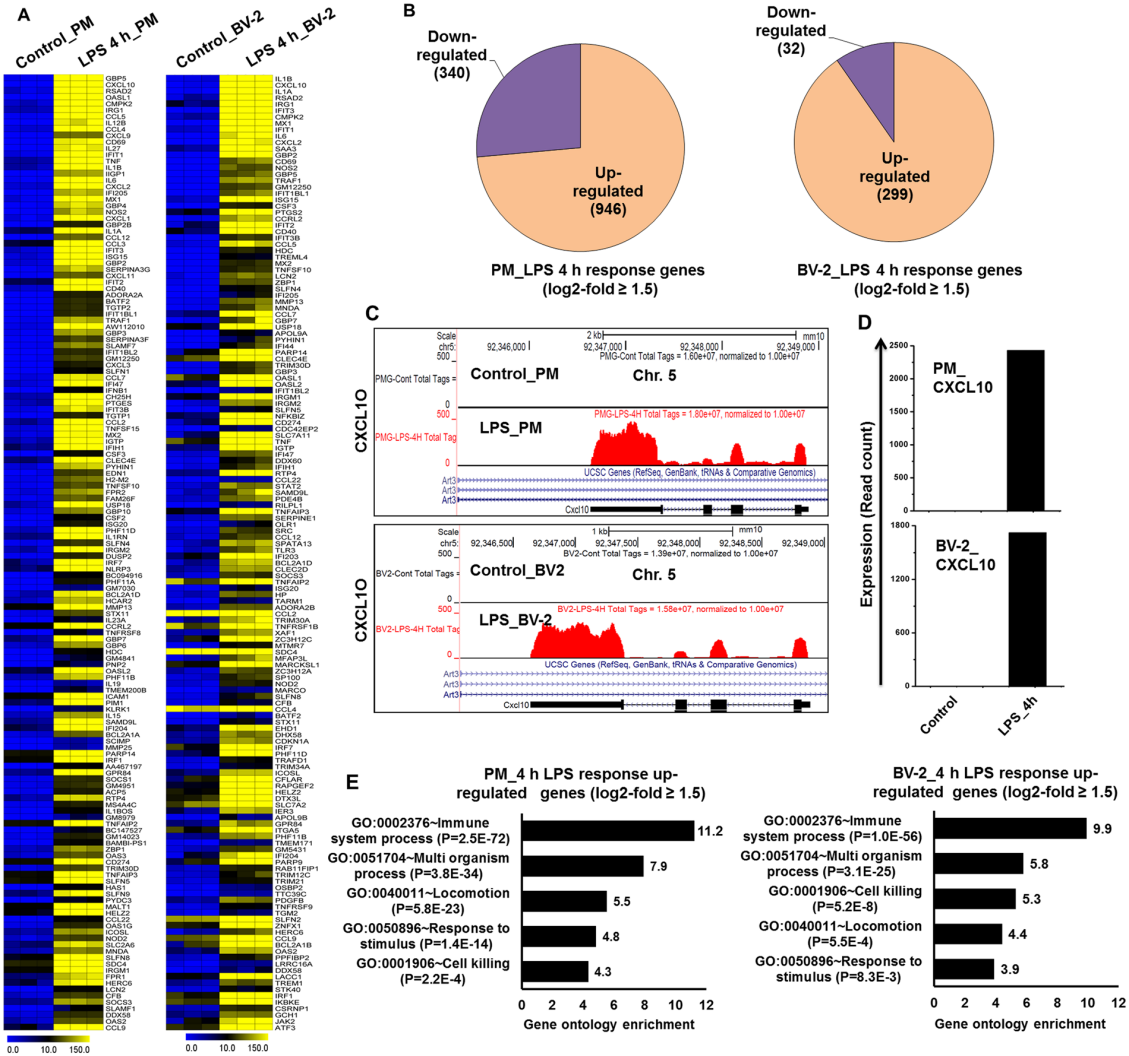
RNA-seq analysis reveals LPS-induced inflammatory response-related genes and their downstream effectors in PM and BV-2 microglial cells. (**A**) A heat map representing the top 150 inflammatory genes in PM and BV2 microglial cells that were up-regulated by LPS stimulation in the RNA-seq gene expression data (*P* ≤ 0.01, and log_2_-fold change ≥1.5). PM and BV-2 microglial cells are compared to the control. Each row shows the relative expression level for a single gene, and each column shows the expression level of a single sample. Heat maps were generated with the Multi Experiment Viewer (version 4.8) software. (**B**) A Venn diagram displaying the number of inducible or repressible genes in PM and BV2 microglial cells. (**C**) UCSC Browser images representing normalized RNA-seq read densities in PM and BV2 microglial cells. (**D**) Transcript abundance (in read count) was evaluated using RNA-seq in LPS-induced PM and BV-2 microglial cells. (**E**) GO analysis of the functional annotations that were associated with up-regulated genes at 4-h after LPS stimulation in the PM and BV-2 microglial cells.

RNA-seq analysis revealed a total of 331 and 1286 genes that were differentially expressed upon stimulation with LPS (increased or decreased in expression with a log_2_-fold change ≥1.5, *P* ≤ 0.01) in BV-2 microglial cells and PM cells, respectively. Of these, 299 and 946 genes were up-regulated, and 32 and 340 genes were down-regulated 4-h after LPS treatment in BV-2 microglial cells and PM, respectively (Fig. 1B). In a previous study, we compared the transcriptomes of BV-2 microglial cells and PM following LPS stimulation and showed that PM reacted stronger to LPS and that therefore, a much larger number of transcripts were altered in PM than in BV2 cell lines^17^. The following inflammatory response- and immune response-related genes exhibited the most dramatic induction levels following LPS challenge: (1) cytokines/chemokines (CCL6, CCL8, CX3CL1, CXCL1, CXCL3, CXCL9, CXCL11, CXCL16, IL12B, IL18BOS, IL18BP, IL19, IL23A, IL27, IRAK1BP1, SOCS1, TNFSF11A, and TNFSF15), (2) interferon-related genes (IRGs) (GBP2B, GBP4, GBP9, GBP10, GBP11, IFI44I, IFIH1, and IFNB1), and (3) one prostaglandin-related gene (PTGS2) (Fig. 1A,C,D). To classify these genes according to biological processes and molecular gene ontology (GO) functions, we categorized these genes using the DAVID (Database for Annotation, Visualization and Integrated Discovery) Bioinformatics Resources^20, 21^ through classification into GO categories (FDR 0.05) based on biological process (BP) and molecular function (MF) as well as KEGG (Kyoto Encyclopedia of Genes and Genomes) pathways. Up-regulated genes were mainly involved in immune system processes and multi-organism processes. Other pathways, such as cell killing, locomotion and responses to stimulus, were also identified in the differentially expressed gene analysis at 4-h after LPS stimulation in the PM and BV-2 microglial cells (Fig. 1E). Down-regulated transcripts affected several developmental processes and GTPase regulator activity in the PM (Supplementary Figure S2). As the down-regulated genes were not associated with inflammation, only the up-regulated genes were further studied.

### GSK-J4 exerts a selective effect on LPS-dependent transcriptional responses in PM and BV-2 microglial cells

**Figure 2:**
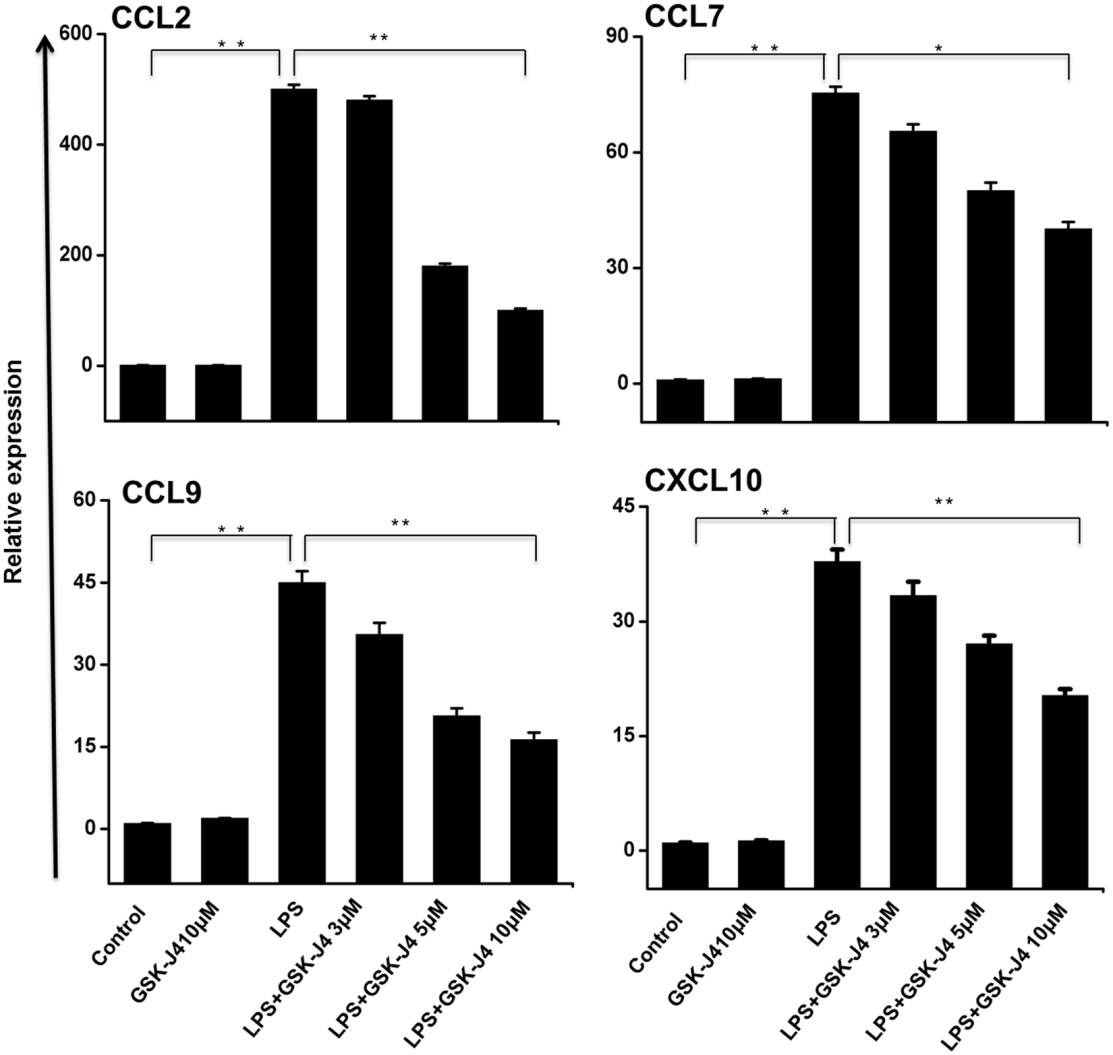
Inhibitory effect of GSK-J4 on LPS-induced BV-2 microglial cells. BV-2 microglial cells were treated with different concentrations of GSK-J4 for 4-h, followed by treatment with LPS (10 ng/ml). Inflammatory genes were significantly down-regulated in cells treated with GSK-J4 compared to untreated cells (*P < 0.01 and **P < 0.001) at the indicated times. Gene expression was normalized to GAPDH transcript levels. The data represent three independent biological experiments. The values are the mean ± SD of triplicate wells.

**Figure 3:**
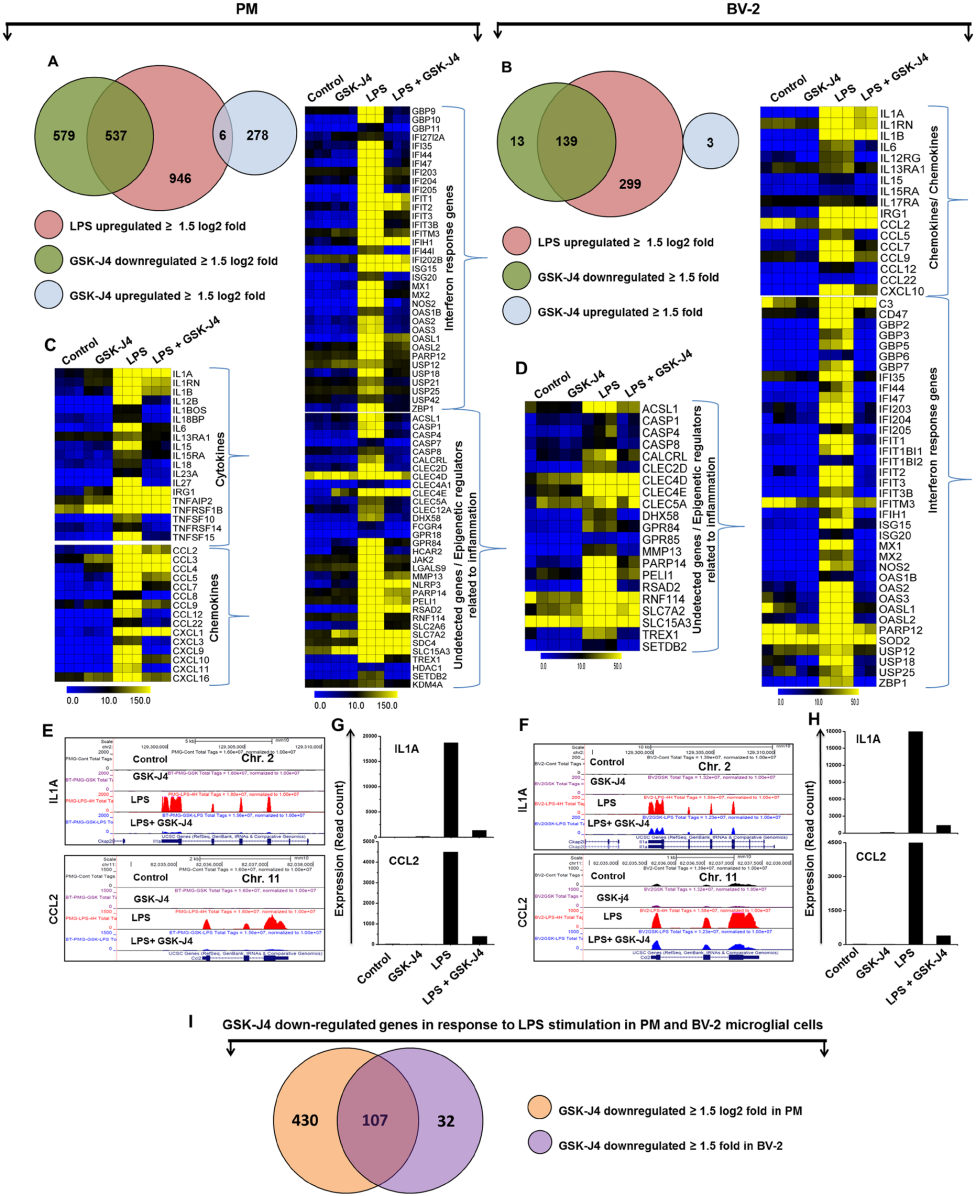
GSK-J4 suppresses a specific subset of LPS-inducible genes in PM and BV-2 microglial cells. (**A**,**B**) A Venn diagram displaying the number of LPS-inducible genes that were suppressed or up-regulated with GSK-J4 treatment at 4-h after LPS stimulation in the PM and BV-2 microglial cells respectively. (**C**,**D**) Heat map representation depicting the positive regulators of inflammatory genes (cytokines, chemokines, IRGs, and undetected genes related to inflammation) that were selectively down-regulated by GSK-J4 at 4-h after LPS stimulation in PM (*P* ≤ 0.01, and log_2_-fold change ≥1.5) and BV-2 microglial cells (*P* ≤ 0.01, and fold change ≥1.5) experiments, respectively. Heat maps were generated with the Multi Experiment Viewer (version 4.8) software. (**E**,**F**) UCSC Browser images representing the normalized RNA-seq read density in GSK-J4-down-regulated inflammatory genes at 4-h in the LPS-induced PM and BV-2 microglial cells compared with controls, respectively. (**G**,**H**) Transcript abundance (in read count) was evaluated using RNA-seq in GSK-J4-down-regulated inflammatory genes at 4-h in the LPS-induced PM and BV-2 microglial cells, respectively. (**I**) The area of overlap indicates the number of unique or shared GSK-J4 down-regulated genes after 4-h of LPS stimulation in PM and BV-2 microglial cells.

To determine whether GSK-J4 is also a broad-spectrum, anti-inflammatory agent, we assessed its efficacy as an immunomodulatory drug that could counter microglia-mediated inflammation. We first examined whether GSK-J4 could alter the expression of inflammation-related genes in microglial cells. As shown in Fig. 2, GSK-J4 inhibited LPS-induced inflammatory genes in a dose-dependent manner while having minimal effect on cell viability (data not shown). Indeed, we observed that 10 μM GSK-J4, a dose used in previous publications^10^, led to a marked reduction in inflammatory gene expression in BV-2 microglial cells (Fig. 2). Based on these results, we chose a concentration of 10 μM GSK-J4 for further experimentation. We then exposed the PM and BV-2 microglial cells to the following four different conditions: (1) untreated, (2) GSK-J4, (3) LPS, and (4) GSK-J4 + LPS. In contrast to the effect of LPS, the GSK-J4 + LPS-treated cells had a substantially less pronounced effect on gene expression and resulted in the down-regulation of 537 and 139 of the LPS-inducible genes at 4-h in PM (*P* ≤ 0.01, and log_2_-fold change ≥ 1.5) and BV-2 microglial cells (*P* ≤ 0.01, and fold change ≥ 1.5), respectively (Fig. 3A,B). GSK-J4 suppressed the expression of key LPS-inducible inflammation- and immunity-related genes, including the CCL2, CCL7, CCL9, CCL12, CXCL10, IL1A, IL1B, IL1RN, IL6, and IL15,TFs; Interferon regulatory factor 7 (IRF7); IRF9; signal transducer and activator of transcription 1 (STAT1); STAT2; and NFXL1 (Fig. 3A–H). To further characterize the GSK-J4 down-regulated genes, we investigated the levels of expression of IRGs. These IRGs included factors known to be involved in antiviral responses such as GBP2, GBP3, GBP5, GBP7, GBP9, GBP10, GBP11, the IFN-induced protein with tetratricopeptide (IFIT) family gene IFIT1 (p56), IFIT2, IFIT3, IFIT3B, IFIH1, IFI35, IFI44, IFI47, IFI203, IFI204, IFI205, IRGM1, IRGM2, IRF3-dependent gene ISG15^22^, ISG20, MX1, MX2, OASL1, OASL1B, OASL1G, USP12, USP18, USP21, USP25 and ZBP1 (Fig. 3C,D). In addition to differentially expressed chemokines/cytokines and IRGs, annotation of the RNA-seq data also revealed approximately 21 and 30 previously unidentified genes that were significantly down-regulated by GSK-J4 upon LPS-treatment in PM and BV-2 microglial cells, respectively (Fig. 3C–H), that were important in inflammatory diseases. Interestingly, inhibition of the other inflammation- and immunity-related genes, such as CXCL2, CSF1, CCRL2, DUSP16, IFNB1, IGSF6, IGSF8, IL17RA, JUNB, TNF-α, TNIP3, TNFAIP3 TNFSF9, REL, and RELB, was marginally affected or unaffected by GSK-J4 (Supplementary Figure S3), suggesting that the GSK-J4-treated, LPS-inducible gene expression profile was highly selective.

### GSK-J4 regulates common and unique transcriptional programs between PM and BV-2 microglial cells

To further investigate the common and unique GSK-J4 down-regulated genes between the LPS-treated PM and BV-2 microglial cells, we again used RNA-seq data to compare the GSK-J4 down-regulated transcriptome of BV-2 microglial cells with PM. Using a similar approach (see above), we compared the GSK-J4 down-regulated transcripts in LPS-treated BV-2 microglial cells with that of PM. Differential expression analysis clearly revealed that GSK-J4 down-regulated a unique gene set in response to LPS stimulation in PM and BV-2 microglial cells (Fig. 3I) suggesting a substantial number of dissimilarities between the two cell types. GSK-J4 down-regulated 430 genes in PM cells that are not common to BV-2 microglial cells. In contrast, GSK-J4 down-regulated 32 genes in BV-2 microglial cells that are not common to PM cells (Fig. 3I). The GSK-J4 down-regulated unique and common gene sets for PM and BV-2 microglial cells are presented in Tables 1, 2 and 3. Of the GSK-J4 down-regulated genes, BV-2 microglial cells and PM shared 107 genes following LPS treatment (Fig. 3I). Importantly, this technology allowed us to identify several specific GSK-J4 down-regulated gene families involved in immune responses that were uniquely altered in LPS-treated PM cells. The major GSK-J4 down-regulated genes in PM only included cytokines/chemokines (CCL3, CCL4, CCL5, CCL8, CCL22, CXCL1, CXCL3, CXCL9, CXCL11, CXCL16, IL1BOS, IL1RN, IL12B, IL13RA, IL15RA, IL15, IL18, IL18BP, IL23A, and IL27) and IRGs (GBP2B, GBP4, GBP6, GBP9, GBP10, GBP11, IFI35, IFI44I, IFI202B, and IFITM3) (Table 1). These data suggest that GSK-J4 down-regulated a unique set of genes that is distinct from that of BV-2 microglial cells and may offer new potential GSK-J4 molecular targets in LPS-induced microglial activation.

**Table 1:** Top 50 GSK-J4 down-regulated unique genes in LPS treated PM.

Gene symbol	Down regulated genes (Log2 fold change)	*P*-value
CXCL9	−8.62	2.0E-26
IL27	−7.80	6.0E-48
IIGP1	−7.26	8.1E-43
CXCL11	−7.00	1.3E-45
GBP4	−6.94	2.5E-65
SLFN1	−6.94	7.5E-13
IL12B	−5.77	1.4E-40
TGTP2	−5.76	2.6E-28
TNFSF15	−5.74	1.1E-171
TGTP1	−5.64	5.0E-30
CH25H	−5.53	0.0E + 00
IL1BOS	−5.40	5.8E-19
FPR2	−5.26	1.1E-117
SLAMF7	−5.24	4.3E-76
CCL8	−5.21	2.1E-16
GBP9	−5.15	5.7E-299
PNP2	−5.04	6.5E-11
XKR8	−4.85	4.5E-81
GBP2B	−4.85	3.1E-19
SLAMF1	−4.74	2.9E-14
OAS1G	−4.74	8.5E-62
CD40	−4.73	3.0E-285
CXCL3	−4.71	2.9E-25
GPR18	−4.68	7.8E-16
ADORA2A	−4.59	1.6E-22
PTGIR	−4.57	2.0E-27
KLRK1	−4.45	1.5E-10
TRIM21	−4.45	8.1E-146
CLIC5	−4.32	1.3E-07
OAS3	−4.27	3.7E-105
CCL5	−4.18	5.1E-154
IL18	−4.10	2.2E-49
IFI35	−4.08	5.4E-156
GBP11	−4.08	1.8E-13
NLRC5	−4.07	0.0E + 00
LACC1	−4.03	2.6E-267
FPR1	−4.02	1.2E-227
IL15	−4.01	6.0E-166
H2-M2	−3.93	3.2E-85
IL18BP	−3.72	1.3E-23
SOCS1	−3.72	3.3E-61
PTGES	−3.70	4.5E-247
ADAMTS4	−3.69	1.1E-91
ZFP811	−3.66	8.7E-18
NOD1	−3.64	5.4E-130
MLKL	−3.63	7.4E-23
CSF2	−3.61	8.1E-14
HAS1	−3.60	3.0E-20
OAS1A	−3.52	1.9E-172
OASL1	−3.51	1.8E-59

**Table 2:** GSK-J4 down-regulated unique genes in LPS treated BV-2 microglial cells.

Gene symbol	Down regulated genes (Fold change)	*P*-value
OLR1	−5.04	1.32E-11
HP	−3.94	4.78E-21
RILPL1	−3.76	4.28E-11
KCNA3	−3.6	6.3E-06
FAM49A	−3.52	6.74E-08
APOL9B	−3.4	4.73E-08
PILRA	−3.28	8.12E-11
PID1	−3.06	1.11E-08
APOL9A	−2.98	1.13E-16
SERPINB6B	−2.78	9.88E-08
LRRC16A	−2.64	7.16E-07
SPATA13	−2.26	1.67E-18
THBS1	−2.26	0.000034
LRRC25	−2.18	3.8E-10
NOTCH1	−2.14	1.14E-11
RHOQ	−2.06	5.14E-09
GCNT2	−2.04	9.48E-08
TRIM12A	−2.02	5.18E-08
NEURL3	−2.02	7.88E-12
RAI14	−1.98	7.87E-12
TREM1	−1.94	1.08E-14
AI607873	−1.86	2.32E-13
MFAP3L	−1.86	5.64E-14
IGSF6	−1.78	1.63E-14
DAAM1	−1.72	1.31E-06
MAP3K8	−1.72	1.18E-08
MID1	−1.68	2.97E-05
TET2	−1.62	7.81E-10
ITGA5	−1.56	8.74E-21
PRDM1	−1.54	7.19E-05
DUSP16	−1.52	3.71E-09

**Table 3:** Top 50 GSK-J4 down-regulated common genes in LPS treated PM and BV-2 microglial cells.

Gene symbol	PM_Down regulated genes (Log2 fold change)	*P*-value	BV-2_Down regulated genes (Fold change)	*P*-value
CD69	−7.59	2.3E-48	−5.96	2.7E-33
IFIT1BL1	−7.11	2.6E-28	−6.60	9.3E-32
NOS2	−6.89	3.7E-55	−1.84	3.5E-31
HDC	−6.87	1.7E-18	−3.02	1.7E-23
TNFSF10	−6.81	1.4E-63	−6.02	5.2E-24
MX1	−6.75	8.5E-206	−4.72	3.6E-53
BATF2	−6.49	1.0E-28	−1.84	4.7E-08
SLFN4	−6.46	1.1E-56	−6.20	6.4E-19
CCL12	−6.37	6.0E-42	−5.08	1.0E-17
IFI205	−6.01	2.7E-50	−2.72	1.9E-16
IGTP	−5.98	5.4E-04	−2.40	6.2E-43
IFI47	−5.55	2.7E-199	−2.96	4.8E-25
IFIT3	−5.27	4.8E-285	−3.44	3.1E-71
CMPK2	−5.22	5.9E-04	−2.48	7.6E-139
IFI44	−5.19	3.4E-236	−5.18	1.5E-16
IL1B	−4.97	3.2E-63	−2.86	1.6E-128
PHF11D	−4.88	1.5E-213	−2.80	5.0E-22
SLFN9	−4.86	7.3E-266	−2.82	1.5E-05
IRGM2	−4.74	5.7E-228	−1.64	3.4E-23
IRF7	−4.65	7.1E-83	−1.34	5.9E-18
USP18	−4.65	2.1E-102	−2.10	3.3E-57
IFIT2	−4.56	5.7E-83	−4.02	2.1E-33
SLFN5	−4.55	4.9E-100	−5.28	2.2E-15
IL6	−4.51	2.1E-99	−3.28	5.1E-45
CLEC2D	−4.51	5.2E-04	−2.86	9.9E-27
IFIT3B	−4.49	2.9E-128	−4.48	5.5E-26
SLFN8	−4.47	3.2E-06	−3.08	6.3E-12
TRIM30D	−4.45	1.9E-93	−4.24	3.9E-21
LCN2	−4.41	3.7E-22	−2.12	2.8E-27
IFI204	−4.40	5.9E-44	−2.62	3.5E-08
GBP2	−4.38	1.4E-227	−1.60	7.0E-48
ISG20	−4.30	1.4E-31	−1.82	4.7E-12
TLR3	−4.22	4.6E-46	−4.88	4.3E-13
MX2	−4.22	5.5E-150	−3.26	2.2E-24
GBP5	−4.17	1.3E-185	−2.10	5.1E-31
H2-T24	−4.10	1.1E-124	−2.46	2.5E-08
ZBP1	−4.10	8.6E-160	−2.12	3.0E-29
GBP7	−4.10	3.3E-302	−3.24	3.6E-19
CSF3	−4.06	5.8E-17	−3.02	4.8E-25
CXCL10	−3.90	1.2E-47	−2.70	1.9E-109
OASL2	−3.86	5.6E-57	−1.72	1.4E-45
SETDB2	−3.84	5.2E-96	−2.12	3.9E-09
STAT1	−3.81	9.2E-57	−1.84	2.0E-23
CCL7	−3.79	3.3E-128	−5.20	2.1E-57
RSAD2	−3.65	2.2E-04	−2.14	1.5E-116
MITD1	−3.64	7.1E-62	−2.72	5.2E-04
IRGM1	−3.62	3.2E-14	−2.08	1.5E-59
IRG1	−3.58	1.2E-08	−1.40	1.5E-156
PARP14	−3.56	1.9E-07	−1.94	8.8E-24

### Differential expression of TFs and signaling pathways modulated through GSK-J4 in LPS-induced PM and BV-2 microglial cells

**Figure 4:**
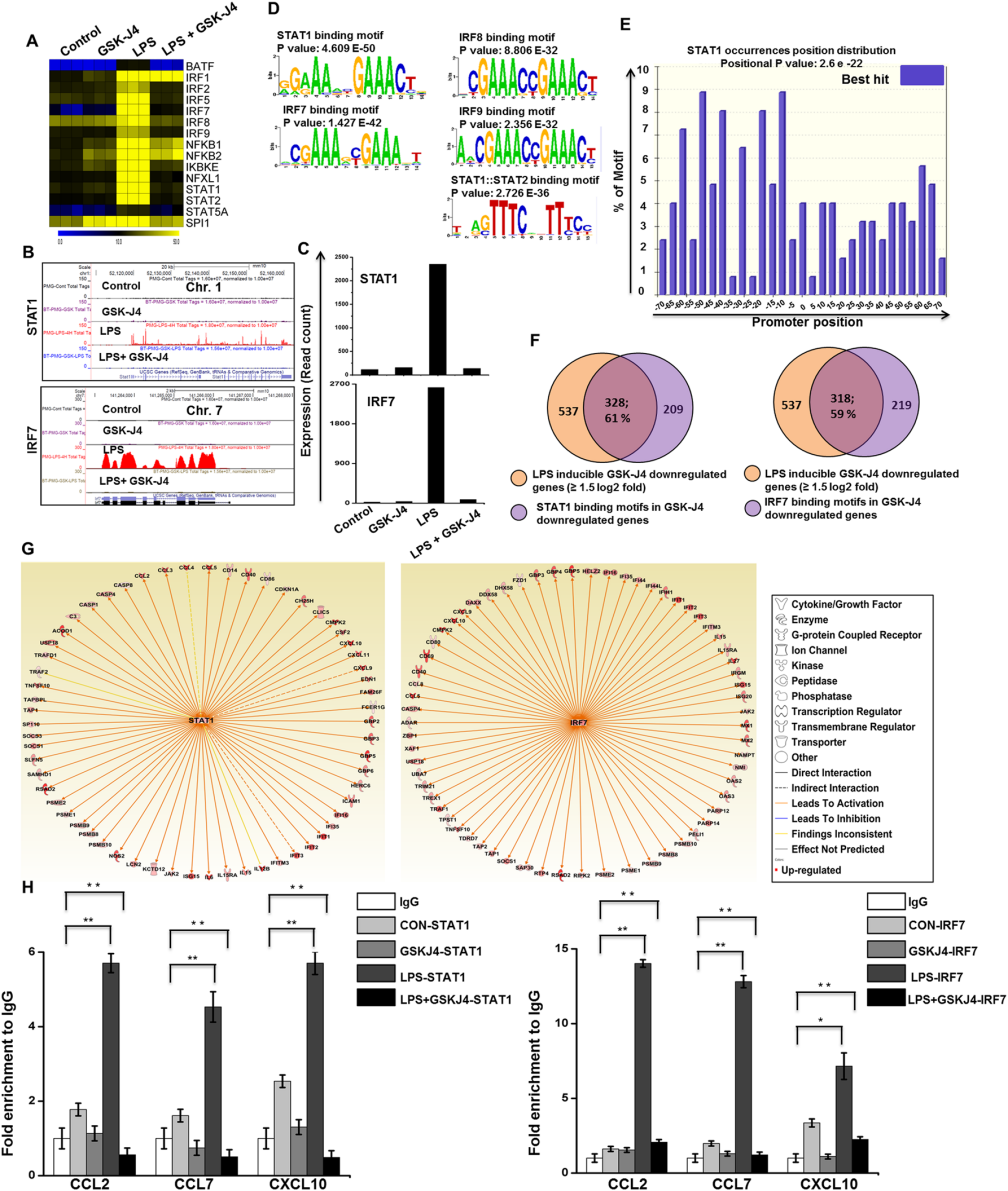
Inhibitory effect of GSK-J4 on LPS-induced key TFs in PM. (**A**) A heat map representation of TF expression levels that were selectively down-regulated (*P* ≤ 0.01, and log_2_-fold change ≥1.5) by GSK-J4 at 4-h after LPS stimulation, from three independent PM experiments. Heat maps were generated with the Multi Experiment Viewer (version 4.8) software. (**B**) UCSC Browser images representing the normalized RNA-seq read density in GSK-J4-down-regulated inflammatory genes at 4-h in the LPS-induced PM compared with controls. (**C**) Transcript abundance (in read count) was evaluated using RNA-seq in GSK-J4-down-regulated TFs at 4-h in the LPS-induced PM. (**D**,**E**) Patterns of TF motif enrichment within the promoters of the GSK-J4-down-regulated genes (*P* ≤ 0.01, and log_2_-fold change ≥1.5) in LPS-induced PM. (**F**) Venn diagrams of GSK-J4-down-regulated genes associated with STAT1 and IRF7 at 4-h in LPS-induced PM. (**G**) The activity of highly connected positive regulators of the inflammatory genes STAT1 and IRF7 led to the activation of this network, as assessed using the IPA molecule activity predictor in GSK-J4-down-regulated genes in PM. (**H**) ChIP assay to determine the presence of STAT1 and IRF7 at selected genes. The ChIP-enriched samples were analyzed using quantitative PCR with selected genes primers. STAT1 and IRF7 binding was increased following LPS exposure, though a reduced presence of STAT1 and IRF7 binding was shown at the promoters of the CCL2, CCL7, and CXCL10 genes in GSK-J4-treated BV-2 microglial cells. The graphs represent the mean fold values of enrichment relative to the IgG control from three independent experiments. *P < 0.01 and **P < 0.001 compared with the control.

To further investigate whether the effect of GSK-J4 altered the key TFs associated with inflammation, we examined multiple families of TFs that were identified after 4-h of LPS stimulation in PM and BV-2 microglial cells. The annotation of the RNA-seq data also revealed that in the presence of LPS stimulation, GSK-J4 also altered the expression of some key TFs, with a log_2_-fold change ≥1.5 and *P* ≤ 0.01 cut-off values (Fig. 4A and Supplementary Figure S4A). These TFs, including IRF and STAT, play important roles in neuroinflammatory diseases^23, 24^. The following TF families exhibited the most dramatic suppression levels following the GSK-J4 challenge: (1) the IRF group of TFs (IRF7 and IRF9); (2) the STAT group of TFs (STAT1 and STAT2); and (3) the other important group of TFs (NFXL1) (Fig. 4A–C and Supplementary Figures S4A–C). Interestingly, IRF1, IRF8, NF-kB1, NF-kB2, and ATF3 were marginally expressed or were unaffected after GSKJ4 treatment upon LPS stimulation (Fig. 4A and Supplementary Figure S4A), suggesting that GSK-J4 suppression of TF expression is highly selective in PM and BV-2 microglial cells. Next, we conducted a TF motif analysis to assess the GSK-J4-suppressed gene expression in PM and BV-2 microglial cells. We used the Pscan software tool^25^ to perform an *in silico* computational analysis of over-represented *cis*-regulatory elements within the 5′-promoter regions of coordinately regulated genes. Applying this score to the gene promoters suppressed by GSK-J4 treatment at 4-h in response to LPS stimulation revealed that the putative binding sites for STAT1, STAT1-STAT2, IRF7, and IRF9 were significantly enriched (Fig. 4D,E and Supplementary Figures S4D,E). Next, we analyzed how many down-regulated genes contain a STAT1 and IRF7 binding motif in the promoter sequence. Interestingly, among down-regulated genes we found a significant percentage of the cytokines and chemokines as well as IRGs had a STAT1 (328/537; 61%) and IRF7 (318/537; 59%) binding motif in the promoter region (from −950 bp to +50 bp), these data are summarized in Fig. 4F and Supplementary Table S1, for PM cells. We also found a significant percentage of STAT1 and IRF7 binding motifs in the promoter region of the cytokines and chemokines as well as IRGs in BV-2 microglial cells (Supplementary Figure S4F and Supplementary Table S1). In addition to TF motif analysis, we also applied IPA software^26^ to identify the target genes that were directly or indirectly activated by the identified TFs in response to GSK-J4 treatment. Importantly, the assessment of upstream regulators by IPA similarly revealed that down-regulation of most of the cytokines and chemokines was also directly regulated by the identified TFs, including STAT1 and IRF7 (Fig. 4G, Supplementary Figure S4G and Table 4).

**Table 4:** Leads to activation of inflammatory genes by identified TFs in response to GSK-J4 inhibited LPS induced inflammatory genes in PM and BV-2 microglial cells.

PM		BV-2	
STAT1 predicted to be activated (61 genes) (*p* = 3.27 e-67)	IRF7 predicted to be activated (65 genes) (*p* = *4.16 e-83*)	STAT1 predicted to be activated (29 genes) (*p* = *5.31 e-55*)	IRF7 predicted to be activated (38 genes) (*p* = 2.09E-66)
CCL2, CCL3, CCL4, CCL5, CD14, CD40, CD86, CDKN1A, CH25H, CLIC5, CMPK2, CSF2, CXCL10, CXCL11, CXCL9, EDN1, FAM26F, FCER1G, GBP2, GBP3, GBP5, GBP6, HERC6, ICAM1, IFI16, IFI35, IFIT1, IFIT2, IFIT3, IFITM3, IL12B, IL15, IL15RA, IL6, ISG15, JAK2, KCTD12, LCN2, NOS2, PSMB10, PSMB8, PSMB9, PSME1, PSME2, RSAD2, SAMHD1, SLFN5, SOCS1, SOCS3, SP110, TAP1, TAPBPL, TNFSF10, TRAF2, TRAFD1, USP18, ACOD1, C2, CASP1, CASP4, CASP8	ADAR, CASP4, CCL5, CCL8, CD40, CD69, CD80, CMPK2, CXCL10, CXCL9, DAXX, DDX58, DHX58, FZD1, GBP3, GBP4, GBP5, HELZ2, IFI16, IFI35, IFI44, IFI44L, IFIH1, IFIT1, IFIT2, IFIT3, IFITM3, IL15, IL15RA, IL27, IRGM, ISG15, ISG20, JAK2, MX1, MX2, NAMPT, NMI, OAS2, OAS3, PARP12, PARP14, PELI1, PSMB10, PSMB8, PSMB9, PSME1, PSME2, RIPK1, RSAD2, RTP4, SAP30, SOCS1, TAP1, TAP2, TDRD7, TNFSF10, TPST1, TRAF1, TREX1, TRIM21, UBA7, USP18, XAF1, ZBP1,	CCL2, CCL5, CD274, CD40, CMPK2, CXCL10, GBP3, GBP5, IFIT1, IFIT2, IFIT3, IL15, ISG15, JAK2, MX1, OAS2, RSAD2, TAP1, TNFSF10, USP18, ACOD1, EIF2AK2, GBP2, HERC6, IL6, NOS2, SLFN5, SP110, TRAFD1	ADAR, CCL5, CD40, CD69, CMPK2, CXCL10, DDX58, DHX58, GBP3, GBP5, HELZ2, IFI44, IFIH1, IFIT1, IFIT2, IFIT3, IL15, IRGM, ISG15, ISG20, JAK2, MX1, MX2, OAS2, OAS3, PARP12, PARP14, PELI1, RSAD2, RTP4, TAP1, TNFSF10, TREX1, TRIM21, UBA7, USP18, XAF1, ZBP1

We also hypothesized that STAT1 and IRF7 would also associate with the target gene promoters. Accordingly, we performed chromatin immunoprecipitation (ChIP) studies to map STAT1 and IRF7 occupancy along the target key pro-inflammatory gene promoter regions in LPS-induced BV-2 microglial cells. As shown in Fig. 4H, LPS-induced STAT1 and IRF7 binding and co-treatment with GSK-J4 led to STAT1 and IRF7 binding interference at the promoter site of key pro-inflammatory genes in BV-2 microglial cells. Our results are consistent with a previously published report that showed that STAT1 binds to the promoter region of several inflammatory genes, and that silencing of the JMJD3 gene significantly repressed the expression of STAT-dependent genes^27^. However, we could not identify significant binding sites for STAT3 at the promoter site of GSK-J4-suppressed genes in BV-2 microglial cells and PM. Furthermore, we again performed ChIP studies to map STAT1 and IRF7 occupancy along the GSK-J4 non-target gene promoters. We found that LPS-induced STAT1 and IRF7 binding and co-treatment with GSK-J4 did not lead to a decrease in STAT1 and IRF7 binding at the promoter site of GSK-J4 non-target genes in BV-2 microglial cells (Supplementary Figure S5). Taken together; these findings suggest that STAT1 and IRF7 TFs might be involved in the regulation of GSK-J4-suppressed microglial cell activation.

### Effect of GSK-J4 alone on resting PM cells

We wondered whether the effect of GSK-J4 alone altered the genes associated with inflammation in resting PM and BV-2 microglia cells. To address this point, we examined the genome-wide effect of GSK-J4 alone in resting PM and BV-2 microglia cells. Our results revealed that GSK-J4 alone, in the absence of LPS stimulation, also altered the expression of genes, with a 1.5 log_2_-fold and *P* ≤ 0.01 cut-off value. Surprisingly, genes associated with the LPS response and inflammation (CCL6, CCL8, CX3CL1, CXCL1, CXCL3, CXCL9, CXCL11, CXCL16, IL12B, IL18BOS, IL18BP, IL19, IL23A, IL27, IRAK1BP1, SOCS1, TNFSF11A, and TNFSF15) were unaffected or were expressed insignificantly. A total of 278 and 3 genes (log_2_-fold ≥ 1.5 and *P* ≤ 0.01) were up-regulated in the PM and BV-2 microglia cells, respectively, that were treated with GSK-J4 alone at 4-h (Fig. 3A,B). Notably, we observed that heat shock protein (HSP) 1 A and 1B, metallothionein (MT) 1 and 2, RASD family member 2 (RASD2), etc. genes were up-regulated in GSK-J4 stimulated PM at 4-h (Supplementary Figures S6A–C). HSPs are class of molecular chaperones that function in the regulation of both necrotic cell death and cell survival. Dysregulation of HSP expression has been demonstrated to play critical roles in the pathogenesis of CNS diseases. For example, HSPA1B was induced in the CNS of MS patients and experimental autoimmune encephalomyelitis (EAE) animals, and it is believed that the inhibition of HSPA1B might be a therapeutic target for EAE in MS patients^28, 29^. In contrast, other studies showed that overexpression of HSPA1B may have a potential role against brain ischemia via an anti-inflammatory mechanism^30, 31^. In addition to HSPA1B, HSPA1A overexpression is likely to be responsible for the protection seen in ischemia^32^. MT-1/MT-2 has been implicated in a wide array of pathological conditions in the brain and in neurodegenerative diseases. In particular, MT-1/MT-2 isoform expression was up-regulated in animal models of MS^33^. RASD2 plays a critical role for the selective toxicity in HD and is suggested to be a potential new target for HD^34, 35^. We confirmed with DAVID Bioinformatics Resources GO analysis (FDR 0.05) that GSK-J4-up-regulated transcripts were associated with rhythmic processes, growth, as well as immune system process (Supplementary Figure S6D). A better understanding of the consequences of only GSK-J4-mediated modulation of microglia activation warrants a comprehensive investigation.

### Functional and pathway analyses following GSKJ4 treatment in LPS-induced PM and BV-2 microglia cells

**Figure 5:**
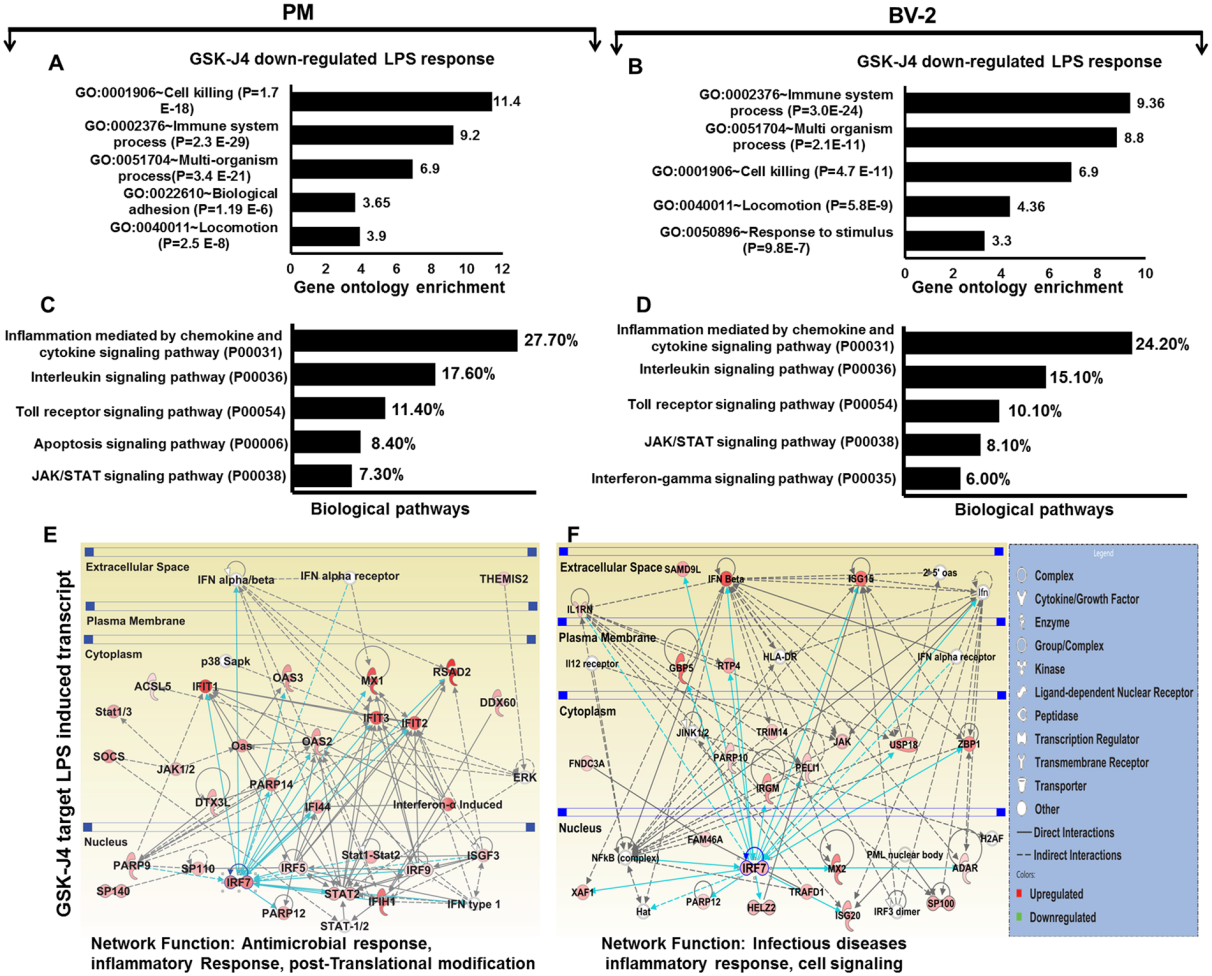
Functional annotation and biological pathways of the GSK-J4 down-regulated genes in PM and BV-2 microglial cells. (**A**,**B**) GO term enrichment analysis for the “biological process” category of the GSK-J4 down-regulated genes in PM and BV-2 microglial cells. The top GO terms are ranked by the GO enrichment. (**C**,**D**) The most highly represented biological pathways of the GSK-J4 down-regulated genes in PM (*P* ≤ 0.01, and log_2-_fold change ≥1.5) and BV-2 microglial cells (*P* ≤ 0.01, and fold change ≥1.5). (**E**,**F**) Ingenuity^®^ Bioinformatics pathway analysis of gene networks displaying interactions between infectious diseases, antimicrobial and inflammatory response-related genes that were down-regulated by GSK-J4 at 4-h after LPS stimulation. Genes in white circles were not in our DEG dataset but were inserted by IPA because these genes are connected to this network. The activity of molecules highly connected to this network, namely IRF7, STAT1 and STAT2 (hubs), was assessed using the IPA molecule activity predictor.

To further explore and functionally classify the GSK-J4 down-regulated genes with LPS stimulation, we again used the DAVID Bioinformatics Resources. Interestingly, we observed that the largest gene groups were involved in the same biological processes, e.g., immune system processes, cell killing, and multi-organism processes (Fig. 5A,B). To determine the potential biological pathways of the GSK-J4-down-regulated genes in the LPS-induced PM and BV-2 microglia cells, we utilized the PANTHER classification system, version 9.0^36^. The major categories of the biological pathways were inflammation mediated by chemokines and cytokines, interleukin and integrin signaling pathways (Fig. 5C,D). To corroborate these functional findings, we analyzed the influence of GSK-J4 on molecular signaling networks in LPS-induced PM and BV-2 microglia cells using IPA^26^. Additionally, GSK-J4 down-regulated gene sets revealed signaling networks related to antimicrobial activity, inflammatory response, and infectious diseases. Particularly, three TFs, including IRF7, STAT1 and STAT2, were identified as the main modulator genes (Fig. 5E,F). Together these data clearly implicate that GSK-J4 may limit the inflammatory response in microglial cells.

### Confirmation of GSK-J4 down-regulated genes by qRT-PCR in PM and BV-2 microglia cells

**Figure 6:**
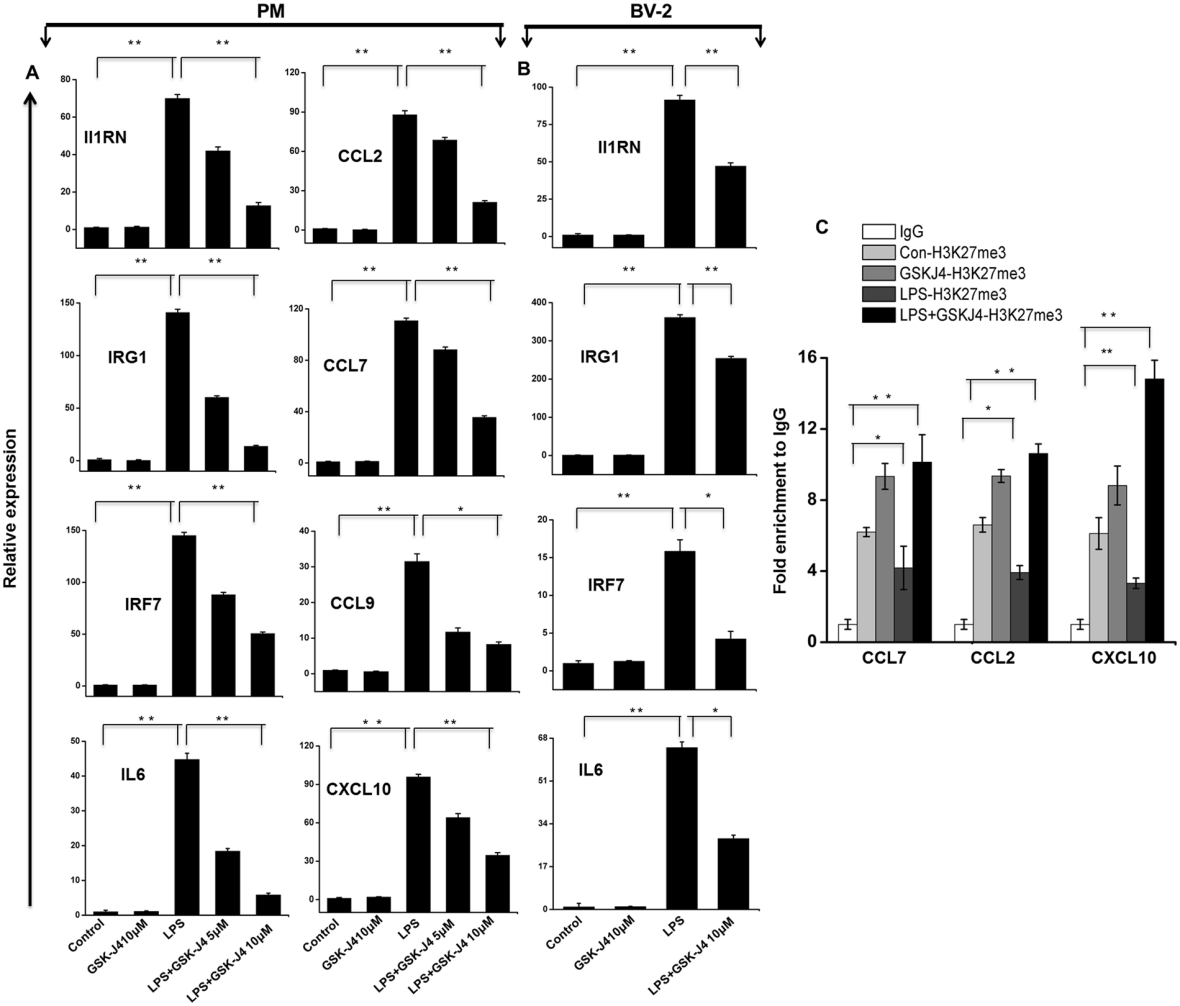
Validation of selected genes by quantitative reverse transcription-polymerase chain reaction in PM and BV-2 microglial cells. (**A**,**B**) The IL1RN, IRG1, IRF7, IL6, CCL2, CCL7, CCL9, and CXCL10 AND IL1RN, IRG1, IRF7, and IL6 genes were significantly down-regulated in the GSK-J4-treated PM and BV-2 microglial cells, respectively. Gene expression was normalized to the GAPDH transcript levels. *P < 0.01 and **P < 0.001 compared with the control. The data represent three independent biological experiments. (**C**) ChIP assay to determine the presence of H3K27me3 at selected genes. The ChIP-enriched samples were analyzed using quantitative PCR with selected gene primers. The H3K27me3 levels were decreased following LPS exposure while an induced presence of H3K27me3 was shown at the promoters of the CCL2, CCL7, and CXCL10 genes in GSK-J4-treated BV-2 microglial cells. The graphs represent the mean fold values of enrichment relative to IgG control from three independent experiments. *P < 0.01 and **P < 0.001 compared with the control.

Several genes that were identified by RNA-seq analysis as differentially regulated were subjected to validation through real-time qRT-PCR using GAPDH as a reference gene. Most were selected for validation according to the distinct effects of GSK-J4 on the LPS-affected genes. To measure gene expression, mRNA was reverse transcribed into cDNA using the Prime Script TM Reverse Transcriptase (Takara Bio Inc., Shiga, Japan) and the qRT-PCR assays were repeated several times using at least 3 mRNA preparations from independent biological experiments. The results are expressed as the fold change relative to the control levels. We found that in almost all cases, there was very good agreement between the RNA-seq and the qRT-PCR results in terms of the direction of change as well as its magnitude. The expression levels of the mRNA of nine genes selected for verification, and the RNA-seq expression pattern was confirmed for eight genes (CCL2, CCL7, CCL9, CXCL10, IL1RN, IRG1, IRF7 and IL6; Figs 2 and 6A,B) in PM and BV-2 microglia cells however, one was non-significant (data not shown) in the qRT-PCR analysis compared with the RNA-seq experiments.

### GSK-J4 inhibits inflammatory genes through H3K27 demethylation in BV-2 microglia cells

To further elucidate the biological relevance of H3K27 demethylation, we performed ChIP studies on GSK-J4 treated BV-2 microglia cells in the presence or absence of LPS. Initially, we targeted the genes from RNA-seq datasets that were significantly down-regulated in the GSK-J4 treated samples but normally up-regulated in the LPS-induced samples (CCL2, CCL7, and CXCL10). We selected the important key pro-inflammatory mediators. Using ChIP assays, we observed that that GSK-J4 prevented the LPS-induced loss of H3K27me3 levels at the promoter site of key pro-inflammatory genes (Fig. 6C), suggesting that global gene expression changes in microglia cells after GSK-J4 treatment by inhibiting H3K27me3 demethylation. We also confirmed the reduced expression of these genes and found that they were down-regulated in the GSK-J4-treated samples (Fig. 2).

### GSK-J4 inhibits pro-inflammatory cytokine expression in mouse brain microglia

**Figure 7:**
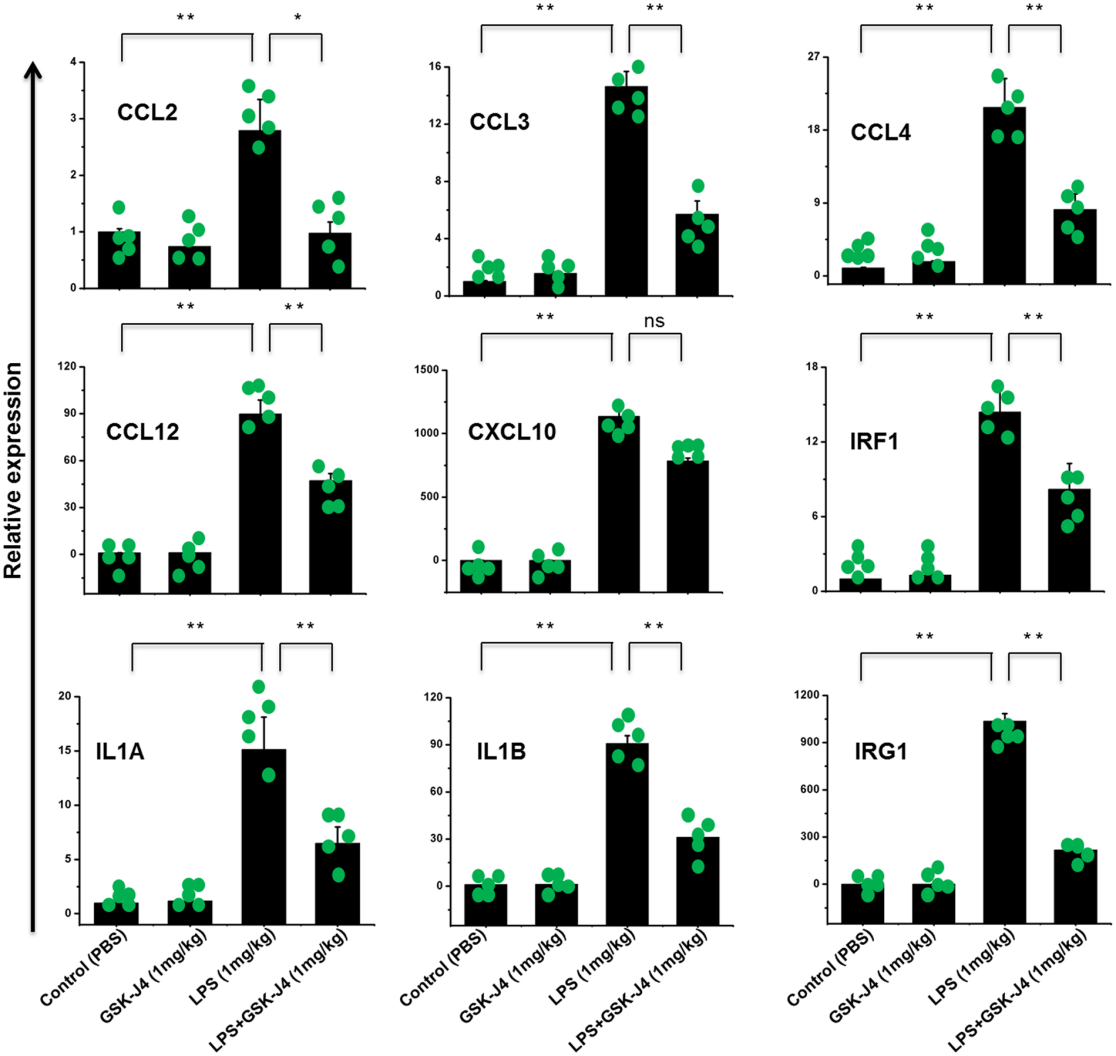
*In vivo* effect of GSK-J4 on pro-inflammatory responses in LPS-challenged mice. ICR mice (n = 5 for each group) were treated with LPS (1 mg/kg) following GSK-J4 injection (1 mg/kg) and brain microglia were collected at 4-h to determine LPS-induced gene expression by qRT-PCR. The CCL2, CCL3, CCL4, CCL12, IRF1, IL1A, IL1B, and IRG1 genes were significantly down-regulated in the GSK-J4-injected adult microglial cells. Gene expression was normalized to GAPDH transcript levels. Each point represents data from an individual mouse, all values shown as mean ± S.E.M. *P < 0.01, **P < 0.001 and ns is non-significant versus all other groups; calculated by two-way ANOVA Tukey’s HSD post-hoc test.

The *in vivo* effect of GSK-J4 on neuroinflammation was examined in an established model of neuroinflammation^37^. After LPS challenge (1 mg/kg), the mice received an intraperitoneal injection of GSK-J4 (1 mg/kg). LPS administration (1 mg/kg) significantly elevated the expression of pro-inflammatory genes in adult microglia (Fig. 7). More importantly, subsequent injection of GSK-J4 significantly suppressed the expression of key LPS-inducible inflammation and immunity-related genes, including CCL2, CCL3, CCL4, CCL12, IRF1, IL1A, IL1B, and IRG1 in the adult microglial cells (Fig. 7). However, it should be noted that the CXCL10 gene was not suppressed by GSK-J4 treatment. Additionally, these pro-inflammatory mediators were not affected by only GSK-J4 injection in adult microglia.

## Discussion

JMJD3 has emerged as a crucial epigenetic regulator governing the assembly of histone demethylation-dependent chromatin complexes that regulate inflammatory gene expression in macrophages^7, 38^. A potent and highly specific JMJD3 inhibitor, GSK-J4 has been shown to disrupt inflammation and cancer^11, 12^. For example, GSK-J4 can inhibit the transcription of a plethora of pro-inflammatory genes in LPS-induced macrophages, including crucial inflammatory genes, such as TNF-α^10^. Additionally, other studies reported even wider prospective applications for GSK-J4, such as in attenuating brainstem glioma or T-cell acute lymphoblastic leukemia (T-ALL), suggesting that GSK-J4 may have anti-inflammatory as well as anti-cancer activities^12^. However, none of these studies addressed the effects of GSK-J4 at the genome-wide expression level in microglial cells. Because previous studies have demonstrated that PM showed a unique molecular expression profile that was different from the profile in BV-2 microglia cells^17, 39^, we examined both PM and BV-2 microglial cells as a model of inflammation, which is one of the major experimental uses of microglia. For the first time, in the present study, we identified a prominent transcriptional response in resting as well as LPS-induced microglial cells after GSK-J4 treatment using RNA-seq analysis. This unbiased profiling approach revealed the importance of GSK-J4 in the regulation of key inflammatory genes involved in the establishment of innate immunity in microglia.

Our RNA-seq data revealed that GSK-J4 repressed the expression of an important subset of pro-inflammatory genes, cytokines/chemokines as well as IRGs, including CCL2, CCL7, CCL9, CCL12, CXCL10, IL1A, IL1B, IL1RN, IL6, IL15, GBP2, GBP3, GBP5, GBP7, GBP9, GBP10, GBP11, IFIT1, IFIT2, IFIT3, IFIT3B, IFIH1, IFI35, IFI44, IFI47, IFI203, IFI204, IFI205, IRGM1, IRGM2, ISG15, ISG20, MX1, MX2, OASL1, OASL1B, OASL1G, USP12, USP18, USP21, USP25 and ZBP1 (Fig. 3C–H). These cytokines/chemokines are also referred to as inflammatory cytokines, and their excessive production has been associated with disease progression and severe inflammation pathologies, including trauma, ischemic injury and MS^40^. CCL2 and CCL7 are potent chemoattractants for monocytes/macrophages and are highly expressed in microglia, astrocytes and other inflammatory cells during MS^41, 42^. Additionally, Conductier *et al*. reported that CCL2 plays a crucial role in neuroinflammatory diseases, and considered it as a target in the treatment of neuroinflammatory disorders^43^. One of the mechanisms to activate microglia is the expression of CD40; it is believed that the induction of CD40 is critical for a productive immune response. Ultimately, increased CD40 expression coupled with the secretion of CCL2 and CCL7 results in exacerbation of neuroinflammation^44, 45^. The expression of CXCL10 or interferon gamma-induced protein 10 (IP-10) has been observed during several neurodegenerative diseases and plays a crucial role in T-cell-mediated inflammation in the CNS^46^. CXCL10 also has a well-established role in inflammatory demyelinating diseases, such as MS, through the destruction of the myelin sheath or neurons by facilitating leukocyte trafficking in the brain^47^. Thus, the down-regulation of numerous inflammatory genes (CCL2, CCL7, CCL9, CCL12, CXCL10, IL1A, IL1B, IL1RN, IL6, IL15, GBP2, GBP3, GBP5, GBP7, GBP9, GBP10, GBP11, IFIT1, IFIT2, IFIT3, IFIT3B, IFIH1, IFI35, IFI44, IFI47, IFI203, IFI204, IFI205, IRGM1, IRGM2, ISG15, ISG20, MX1, MX2, OASL1, OASL1B, OASL1G, USP12, USP18, USP21, USP25 and ZBP1) through GSK-J4 would have great utility in reducing neuroinflammation in the CNS.

It is well-known that IRFs and STATs are important TFs involved in the regulation of inflammation disorders, including neurodegenerative diseases. We showed here that GSK-J4 repressed the expression of an important subset of LPS-inducible TFs, especially IRF7, IRF9, STAT1, and STAT2 (Fig. 4A). STATs have been implicated in several CNS pathologies. Due to its critical role, the STAT family is suggested to be one of the most extensively studied targets in inflammation^34^, and inhibition of STAT activity is likely to function as a potent therapeutic strategy^48^. IRFs are a family of TFs that are involved in inflammatory diseases^49^. Type 1 IRFs have well-characterized pro-inflammatory and anti-viral roles in neuroinflammation. Indeed, IRF7 is a significant regulatory factor in the development of demyelination diseases in the CNS, such as MS and EAE^33^, whereas IRF9 is important in injury-induced type 1 IRF signaling, which regulates inflammatory responses in the CNS^50^. Furthermore, GSK-J4 also inhibited the expression of a wide group of other IRGs in LPS-induced PM and BV-2 microglial cells. Thus, the down-regulation of STAT1, STAT2, IRF7 and IRF9 through GSK-J4 could inhibit neurodegenerative diseases as well as brain inflammation. Finally, the results achieved by the real-time RT-PCR analysis of CCL2, CCL7, CCL9, CXCL10, IL1RN, IRG1, IRF7 and IL6 (Fig. 6A) illustrate the essential down-regulated expression of the abovementioned mRNAs in GSK-J4-treated PM and BV-2 microglial cells when compared to the control. Moreover, ChIP studies confirmed that GSK-J4-down-regulated genes prevented the LPS-induced loss of H3K27me3 as well as interfered with the STAT1 and IRF7 binding at the promoter site of key pro-inflammatory genes in BV-2 microglial cells (Fig. 6D).

One of the most striking features is that GSK-J4 significantly suppressed the expression of previously unidentified inflammatory genes that are induced by LPS in PM and BV-2 microglial cells (Fig. 3C,D). MMPs play a pivotal role in neuroinflammation and neurodegenerative disorders, and MMP13 has been found to be activated in brain tissue^51, 52^. Orphan G protein-coupled receptor 84 (GPR84) expression is restricted to microglia and observed in different pathological conditions, including EAE and endotoxemia^53^. PELI1 plays a critical role in microglia activation during EAE pathogenesis and is suggested to be a potential new target for MS therapy^54^. Accordingly, our current results showed down-regulation of MMP13, GPR84, and PELI1 expression (Fig. 3C,D); the inhibition of these genes by GSK-J4 could underlie the potential benefits in treatment of neuroinflammatory diseases. However, the mechanism by which GSK-J4 inhibits key inflammatory genes requires further study.

In the absence of LPS stimulation, the treatment of PM with GSK-J4 did not have an impact on LPS-induced inflammatory gene expression. It is interesting to note that crucial inflammatory genes, including CXCL2 and TNF-α as well as other inflammatory and immunity-related genes, such as CCRL2, CSF1, DUSP16, IGSF6, IGSF8, IL17RA, IFNB1, JUNB, REL, RELB, TNFAIP3, TNFSF9, and TNIP3, were unaffected by GSK-J4 in PM and BV-2 microglial cells (Supplementary Figure S3). This specificity and anti-inflammatory potential of GSK-J4 was validated using qRT-PCR analysis (data not shown). In contrast, Kruidenier *et al*. demonstrated that GSK-J4 is a potent inhibitor of TNF-α production in macrophages^10^. Importantly, we observed that the prominent TFs STAT1, STAT2, IRF7, and IRF9 were suppressed by GSK-J4, although surprisingly, GSK-J4 had no effect on the master TFs NF-kB (REL and RELB) or AP-1 (ATF-3 and JUNB) in PM. It has been demonstrated that the NF-kB pathway is a critical player in the regulation of TNF-α and CXCL2 expression in macrophages^55, 56^. Furthermore, other reports have shown that both the AP-1 and NF-kB pathways prominently regulate TNF-α gene expression^57^. Therefore, it seems likely that the LPS-induced induction of TNF-α and CXCL2 transcription depends on NF-kB or AP-1, rather than the STAT1, STAT2, IRF7, and IRF9 transcriptional pathways in microglia. Furthermore, we confirmed that GSK-J4 did not lead to a decrease in STAT1 and IRF7 binding at the TNF-α and CXCL2 gene promoters in BV-2 microglial cells (Supplementary Figure S5). A better understanding of the consequences of LPS-induced cytokines/chemokines production modulated by GSK-J4 in microglial cells warrants a comprehensive investigation.

Interestingly, a recent report demonstrated that systemic GSK-J4 treatment decreased the development of EAE in mice^58^. However, the identification of the GSK-J4 molecular target in LPS-induced microglia has not yet been examined using RNA-seq analysis. Collectively, our RNA-seq data raised the possibility that GSK-J4 may play a potential role in the treatment of neuroinflammatory diseases, possibly through its inhibition of microglia and the following inflammatory responses in the CNS. We also showed that GSK-J4 controls important inflammatory gene targets by modulating STAT1, IRF7, and H3K27me3 levels at their promoter sites. Nevertheless, further extensive *in vivo* research is needed to investigate the anti-inflammatory effect of GSK-J4 and its underlying mechanism, which may finally result in the development of effective and safe anti-inflammatory drugs.

## Conclusion

On the basis of RNA-seq data we speculated that GSK-J4 triggered global changes in resting and LPS-induced microglial cells transcriptomes, targeting inflammatory diseases of the CNS. The findings suggest for the first time that GSK-J4 selectively inhibits the expression of several immune and inflammation-related genes, including cytokines, chemokines, interleukins, interferons and TFs, as well as novel inflammatory targets, to exert its anti-inflammatory functions. Moreover, GSK-J4 could be targeted by epigenetic-focused drug discovery, as it may have therapeutic applications in inflammation-mediated neurodegenerative diseases.

## Methods

### Isolation, culture, and stimulation of microglial cells

Mouse BV-2 microglial cells were grown in high-glucose Dulbecco’s modified Eagle’s medium (DMEM) supplemented with 10% fetal bovine serum (FBS) (catalog # 26140; Gibco, Waltham, MA), 100 IU/ml penicillin, and 10 µg/ml streptomycin (catalog # 15140; Invitrogen, Waltham, MA). The cells were maintained in a humidified incubator with 95% air and a 5% CO_2_ atmosphere at 37 °C and JMJD3 inhibitor GSK-J4^10^ were purchased from Tocris (4594; Bristol, UK) and dissolved in dimethyl sulfoxide (DMSO, Sigma- Aldrich, St. Louis, MO) as a 20 mM stock solution; the stock solution was diluted in DMEM for experiments. The cells were incubated with LPS (10 ng/ml) for the specified times under normal culture conditions. LPS (L6529; strain 055:B5) were purchased from Sigma-Aldrich, St. Louis, MO. Medium containing the appropriate agents was replaced every other day. PM were isolated from 3-day-old ICR mice as previously described^59^. All experimental protocols were performed in accordance with Institutional Animal Care and Use Committee (IACUC) guidelines and approved by the IACUC committee of Hanyang University (HY-IACUC-2014-0164A and HY-IACUC-2015-0075). Briefly, whole brains of neonatal mice were dissected out of the skull, and blood vessels and meninges were carefully removed. Then, the tissues from whole brains obtained from 12 mice were pooled together, finely minced, and digested using a Neural Tissue Dissociation Kit-Postnatal Neurons (Miltenyi Biotec-130-094-802). Next, the digested cells were passed through a 70-µm nylon cell strainer (BD Bioscience, Franklin Lakes, NJ) and seeded in poly-L-lysine coated T-75 flasks in DMEM/nutrient mixture F-12 (DMEM/F12, 1:1) containing 20% FBS (catalog # 26140; Gibco, Waltham, MA), 100 IU/ml penicillin and 10 µg/ml streptomycin (catalog # 15140) obtained from Invitrogen (Waltham, MA). The cells were maintained in a humidified incubator with a 95% air/5% CO_2_ atmosphere at 37 °C. The medium was changed every 2–3 days. After two weeks in culture, the mixed glial cell cultures were shaken at 150 rpm at 37 °C for 45 min, and the glial cell suspensions were collected from each flask and seeded on poly-L-lysine coated cell culture plates. PM cells were sub-plated and used for further experiments. More than 92% of cells obtained were microglia as quantified by CD11b (rat monoclonal immunoglobulin G2b (IgG2b), clone M1/70.15.11.5, Miltenyi Biotec Inc., Auburn, CA, USA) FACS analysis (Supplementary Figure S1A).

### Animals and microglial isolation

All experimental protocols were conducted in accordance with Institutional Animal Care and Use Committee (IACUC) guidelines and approved by the IACUC committee of Hanyang University (HY- IACUC-2016–0050 and HY-IACUC-2017–0004). Adult mouse microglia were prepared from 5–6-weeks-old ICR mice as previously described^60^ with minor modifications. Briefly, mouse brains (excluding the brain stem but including the cerebellum) were dissected out of the skull, and blood vessels and meninges were carefully removed. Then, the mouse brains were cut into small pieces (1–2 mm), vigorously triturated to obtain single cell suspensions, and digested using a Neural Tissue Dissociation Kit-Postnatal Neurons (Miltenyi Biotec, Germany, 130–094–802). Myelin debris was removed using modified protocols from Miltenyi Biotec’s, Germany (Myelin Removal Beads II, 130–096–731). After myelin removal, cells were stained with PE-conjugated anti-CD11b antibodies (Miltenyi Biotec, Germany, 130–093–634) followed by incubation for 15 minutes with anti-PE magnetic beads. Magnetically tagged CD11b + cells were then isolated using MS columns according to the Miltenyi Biotec MACS protocol. For immunocytochemistry, purified microglial cells were seeded in 4-well plates. The purity of the primary microglia was 90–91% as determined by immunocytochemistry with antibodies against CD11b^61^ (Supplementary Figure S1B,C).

### LPS and GSK-J4 injections

GSK-J4 was dissolved in DMSO as a 10-mM stock solution. In the systemic drug evaluation experiments, each mouse received intraperitoneal injections of PBS or LPS (1 mg/kg), followed by GSK-J4 (1 mg/kg). The control mice received intraperitoneal injections of PBS. Four hours after injection, the animals were euthanized and the brains were dissected and processed as previously described.

### Immunocytochemistry

Microglia cells were seeded onto coverslips in 4-well plates. The cells were washed with PBS, fixed with 4% paraformaldehyde for 15 min and then permeabilized with cold methanol for 5 min. After blocking with 5% BSA in PBS for 1 h, the cells were incubated with primary antibody overnight at 4 °C with CD11b monoclonal antibody (1:200, Abcam, Cambridge, U.K) followed by incubation with an appropriate secondary donkey anti-rabbit IgG antibody (Thec Jackson Laboratory, West Grove, PA) for 1 h at room temperature. After incubation, the cells were washed with PBS three times and then mounted with 4′,6-diamidino-2-phenylindole (DAPI)-containing mounting solutions (Vectashield, Vector Laboratories, Burlingame, CA) and imaged with an immunofluorescence microscope (Nikon, Tokyo, Japan).

### Total RNA isolation and cDNA library preparation for transcriptome sequencing (RNA-seq)

Total RNA was extracted using RNAiso Plus (Takara Bio Inc., Shiga, Japan) and a QIAGEN RNeasy® Mini kit (QIAGEN, Hilden, Germany). PM or BV-2 microglial cells were completely lysed using RNAiso Plus, and then 200 μl of chloroform was added. The tubes were then inverted for 5 min. The mixture was centrifuged at 12,000 × *g* for 15 min at 4 °C, and the upper phase was placed into a new tube. A 600 μl volume of 70% ethanol was added, and the mixture was applied to an RNeasy mini column. The column was washed with wash buffer. To elute the RNA, RNase-free water (30 μl) was added directly onto the RNase mini column, which was then centrifuged at 12,000 × *g* for 3 min at 4 °C. To deplete ribosomal RNA (rRNA) from the total RNA preparations, a RiboMinus Eukaryote kit (Life Technologies, Carlsbad, CA) was used according to the manufacturer’s instructions. RNA libraries were created using a NEBNext® Ultra™ directional RNA library preparation kit for Illumina® (New England Biolabs, Ipswich, MA). The obtained rRNA-depleted total RNA was fragmented into small pieces using divalent cations at elevated temperatures. First-strand cDNA was synthesized using reverse transcriptase and random primers, and second-strand cDNA synthesis was then performed using DNA polymerase I and RNase H. The cDNA fragments were processed using an end-repair reaction after the addition of a single ‘A’ base, followed by adapter ligation. These products were purified and amplified using PCR to generate the final cDNA library. The cDNA fragments were sequenced using an Illumina HiSeq. 2000. Biological triplicate RNA sequencing was performed on independent RNA samples from the LPS & GSKJ4-stimulated PM: control 4-h (3 samples); GSK-J4 4-h (3 samples), LPS 4-h (3 samples), and LPS + GSK-J4 4-h (3 samples) and BV-2 microglial cells: control 4-h (3 samples); GSK-J4 4-h (2 samples), LPS 4-h (3 samples), and LPS + GSK-J4 4-h (2 samples).

### Differentially expressed gene analysis using RNA-seq data

FASTQ files from RNA-seq experiments were clipped, trimmed of adapters, and the low-quality reads were removed by the Trimmomatic^62^ Quality controlled FASTQ files were alignment to *Mus musculus* UCSC mm10 reference genome sequence using the STAR (version 2.5.1) aligner software^63^. To measure differential gene expression, DESeq. 2^64^ with the default parameters was used. A subset of condition-specific expression was defined as showing a log_2_ fold change ≥1.5 and *P* ≤ 0.01 in expression between controls, GSK-J4, LPS, and LPS + GSK-J4 treated samples. The RNA-seq experiments were visualized using HOMER^65^ after custom tracks were prepared for the UCSC Genome Browser (http://genome.ucsc.edu/). The acquired data were deposited in the Gene Expression Omnibus database under dataset accession no. GSE79898, GSE80304 and GSE89817.

### Quantitative real-time RT-PCR (qRT-PCR)

The reverse transcription of the RNA samples was performed as previously described^17^ using 2 µg of total RNA, 1 µl of oligo (dT) primer (per reaction) and a Prime Script 1st strand cDNA Synthesis Kit (Takara Bio Inc., Shiga, Japan). The oligo (dT) primer and RNA templates were mixed and denatured at 65 °C for 5 min and then cooled for 2 min on ice. Prime Script buffer (5x), RTase and RNAse inhibitor were added to the cooled template mixture and incubated for 1 h at 50 °C before an enzyme inactivation step was performed at 70 °C for 15 min. qRT-PCR was performed using SYBR Green PCR Master Mix (Takara Bio Inc., Shiga, Japan) and a 7500 Real-time PCR System (Applied Biosystems, Waltham, MA). Glyceraldehyde-3-phosphate dehydrogenase (GAPDH) was used as an internal control. Complementary DNA samples were diluted 1.5-fold, and qRT-PCT was performed using an ABI-7500 Real-time PCR System (Applied Biosystems, Waltham, MA) with SYBR Premix Ex-Taq II (Takara Bio Inc., Shiga, Japan) according to the manufacturer’s instructions. The reactions were performed in a total volume of 20 µl that contained 0.4 mM of each primer (Supplementary Table S2). Each PCR series included a no-template control that contained water instead of cDNA and a reverse transcriptase-negative control for each gene. Triplicate measurements were performed for all reactions. Different samples were evaluated using 96-well plates in the gene expression experiments, and all samples were analyzed on a single plate for the endogenous control experiments. The results were analyzed using the critical threshold (∆C_T_) methods in the ABI-7500 software program with the Norm finder and geNorm-plus algorithms. The primers were designed using Primer Express software (Applied Biosystems, Waltham, MA).

### Chromatin immunoprecipitation (ChIP) assay

ChIP experiments were performed using a protocol adapted from Upstate. Chromatin from 1 × 10^7^ cells was used for each immunoprecipitation. BV-2 microglial cells were collected and resuspended in digestion buffer (50 mM Tris-Cl, pH 7.6; 1 mM CaCl_2_, 0.2% Triton X-100, 5 mM butyrate, 1X protease inhibitor cocktail, 0.5 mM PMSF), followed by incubation with 0.3 U Micrococcal nuclease (MNase; Sigma-Aldrich, St. Louis, MO) at 37 °C for 5 min. The reaction was stopped with 50 mM EDTA and treated with RIPA buffer for 16 h, followed by incubation with an approximately 3 µg of primary antibody and Dynabeads Protein A beads (Invitrogen) for 16 h at 4 °C. The antibody used was against H3K27me3 (Cell Signaling #9733), STAT1 (Santa Cruz sc-345), IRF7 (Santa Cruz sc-9083) with normal rabbit IgG (Santa Cruz sc-2025) used as a control. The DNA was extracted and purified following Upstate’s instruction and used in regular PCR analysis. The chromatin immunoprecipitates for the proteins and H3K27me3, STAT1, IRF7 marks were analyzed using regular PCR, with one modification: the cDNA was replaced with immunoprecipitated DNA. The relative enrichment levels indicate the fold changes over the IgG control. Primer sequences were CCL2 forward, AGCCAACTCTCACTGAAGCC; CCL2 reverse, GGGTGATATGCTGGGAAGGG; CCL7 forward, GGCCAGCCTCACATTACACT; CCL7 reverse, CTCTGCTCTTCTGGCAGCTC; CXCL2 forward, CCAACCACCAGGCTACAGG; CXCL2 reverse, GCGTCACACTCAAGCTCTG; CXCL10 forward, TCAGAACAGAAGCCGGAAGT; CXCL10 reverse, TCCTTGACCCTGTAACCACAC; TNF-α forward, CCCCAGATTGCCACAGAATC; TNF-α reverse, CCAGTGAGTGAAAGGGACAG.

### Functional annotation and pathways

To functionally annotate the most significant genes, GO analysis was performed using DAVID, version 6.8^20^. GO was analyzed using a modified Fisher’s exact *P* value in the DAVID program. *P*-values less than 0.001 were considered to be greatly enriched in the annotation category. To determine the possible biological pathways involved in the GSK-J4 treated PM and BV-2 microglial cells, a gene classification analysis of the down-regulated genes was performed using the PANTHER classification system version 9.0 (http://www.pantherdb.org), as described previously^36^. Genes from the datasets that were associated with biological pathways in the PANTHER Pathways Knowledge Base were considered for literature analysis.

### Canonical pathway analysis of datasets

An Ingenuity Pathway Analysis (IPA) (Ingenuity Systems, http://www.ingenuity.com, CA) was performed to analyze the most significant canonical pathways in the datasets as previously described^26^. The genes from datasets associated with canonical pathways in the Ingenuity Pathways Knowledge Base (IPAKB) were considered for literary analysis. The significance of the associations between datasets and canonical pathways was measured in the following manner: (1) the ratio of the number of genes from the dataset that mapped to a canonical pathway was divided by the total number of genes that mapped to the same canonical pathway; and (2) Fisher’s exact test for a *P* value indicating the probability that the association could be explained by chance. After uploading the datasets, gene identifiers were mapped to corresponding gene objects, and the genes were overlaid onto a global molecular network in the IPAKB. Gene networks were algorithmically generated based on connectivity.

### Transcription factor binding motif enrichment analysis

NCBI reference sequence mRNA accession numbers were subjected to transcription factor binding motif analysis using the web-based software Pscan^25^. The JASPAR^66^ database of transcription factor binding sequences was analyzed using enriched groups of −950 base pair (bp) sequences to +50 bp of the 5′ upstream promoters. The range −950 to +50 was selected from the range options in Pscan to obtain the best coverage for a −1000 to +50 bp range.

### Statistical analysis

The data were analyzed using Origin Pro 8 software (Origin Lab Corporation, Northampton, MA, USA). Each value is expressed as the mean ± standard error of the mean (SEM). The statistical analyses were performed using SPSS 17.0 software (SPSS Inc., IL, USA). The data were tested using one-way ANOVA, with the exception of the *in vivo* tests that were analyzed by two-way ANOVA-repeated measures followed by Tukey’s HSD post-hoc correction for comparisons. ^*^ *P* < 0.01 and ^**^ *P* < 0.001 were considered significant.

## Electronic supplementary material

Figure S1, S2, S3, S4, S5, S6 and Supporting Information Table 1, and 2
