# Supplementary material for: RNA sequencing reveals resistance of TLR4 ligand-activated microglial cells to inflammation mediated by the selective jumonji H3K27 demethylase inhibitor
Source: Sci Rep. 2017 Jul 26;7:6554. doi: 10.1038/s41598-017-06914-5 (PMC5529413; doi:10.1038/s41598-017-06914-5)
Supplement: Supplementary file 1 — Figure S1, S2, S3, S4, S5, S6 and Supporting Information Table 1, and 2 [file 41598_2017_6914_MOESM1_ESM.doc]

**Title: RNA sequencing reveals resistance of TLR4 ligand-activated microglial cells to inflammation mediated by the selective jumonji H3K27 demethylase inhibitor**

Amitabh Das^a†^, Sarder Arifuzzaman^b†^, Taeho Yoon^c^, Sun Hwa Kim^c^, Jin Choul Chai^c^, Young Seek Lee^c^, Kyoung Hwa Jung ^a^, **, and Young Gyu Chai ^b, c^, *

^a^*Institute of Natural Science & Technology, Hanyang University, Ansan, 15588, Republic of Korea*

^b^*Department of Bionanotechnology, Hanyang University, Seoul, 04673, Republic of Korea*

^c^*Department of Molecular & Life Sciences, Hanyang University, Ansan, 15588, Republic of Korea*

^†^Equal contributors

**
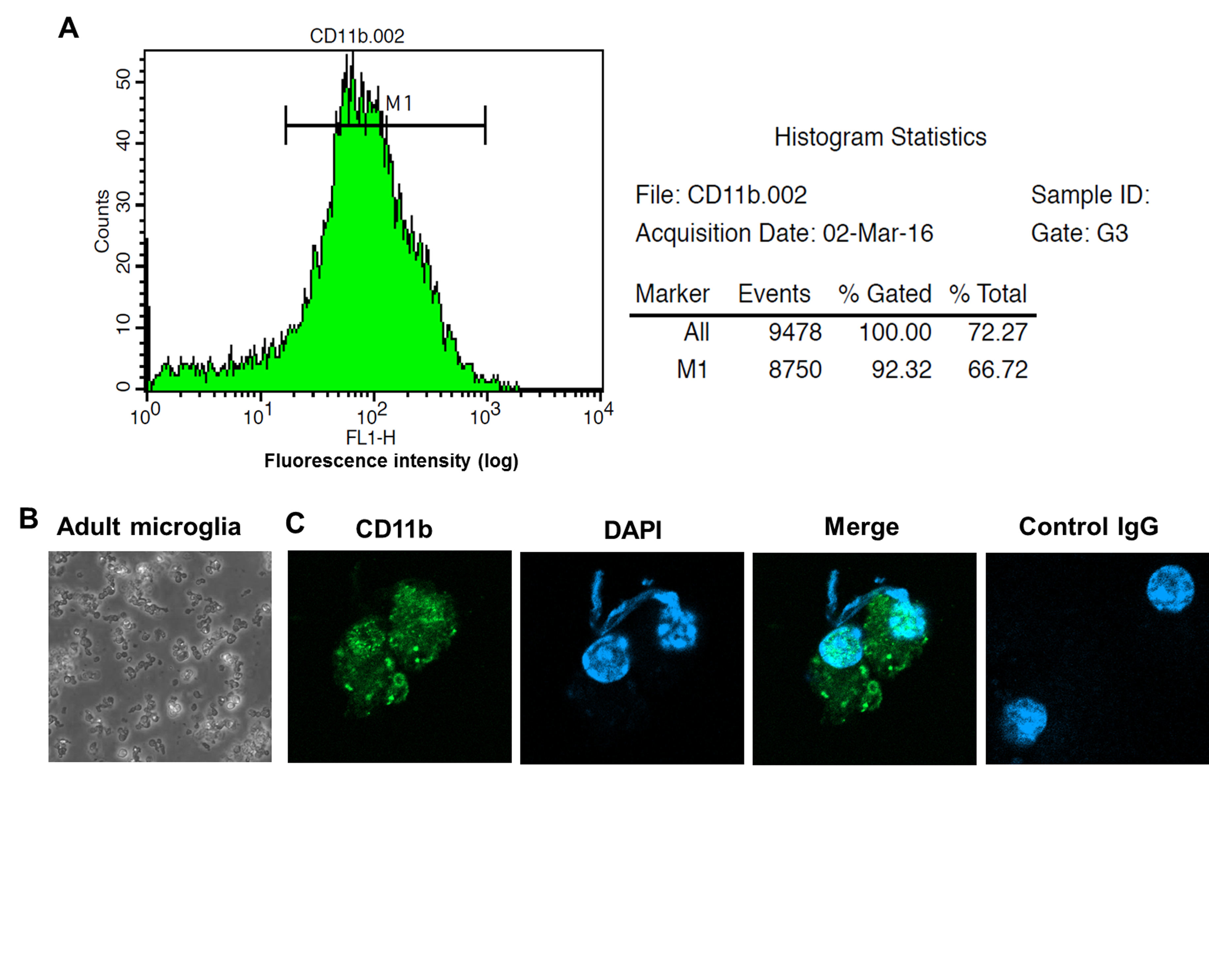
**

**Figure S1 Quantification of CD11b-positive microglial cells.** (A) Microglial purity was assessed using flow cytometry. In total, 92.32% of the cells obtained were PM microglia as quantified by CD11b. The labeled cells are represented by green shaded populations. (B) Photomicrographs are representative images of adult microglia. Images are at 20x magnification. (C) CD11b is expressed on adult mouse microglia. Adult mouse microglia were stained with CD11b or an isotype-matched control followed by incubation with a FITC-labeled secondary antibody and visualization by fluorescence microscopy. The cells were digitally photographed. Data are representative of three independent similar experiments results.

**
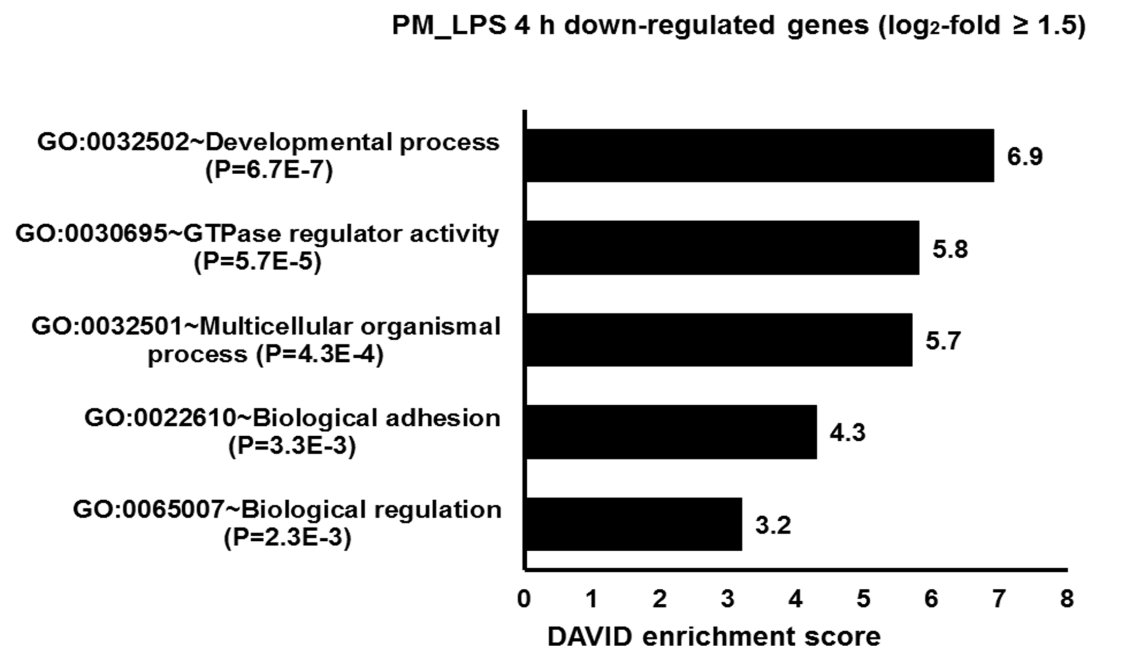
**

**Figure S2 Functional annotations of 4 h LPS-inducible down-regulated genes in PM.** Gene Ontology analysis of functional annotations (biological processes) associated with the top 150 LPS-inducible down-regulated genes at 4 h in PM compared to the control.

**
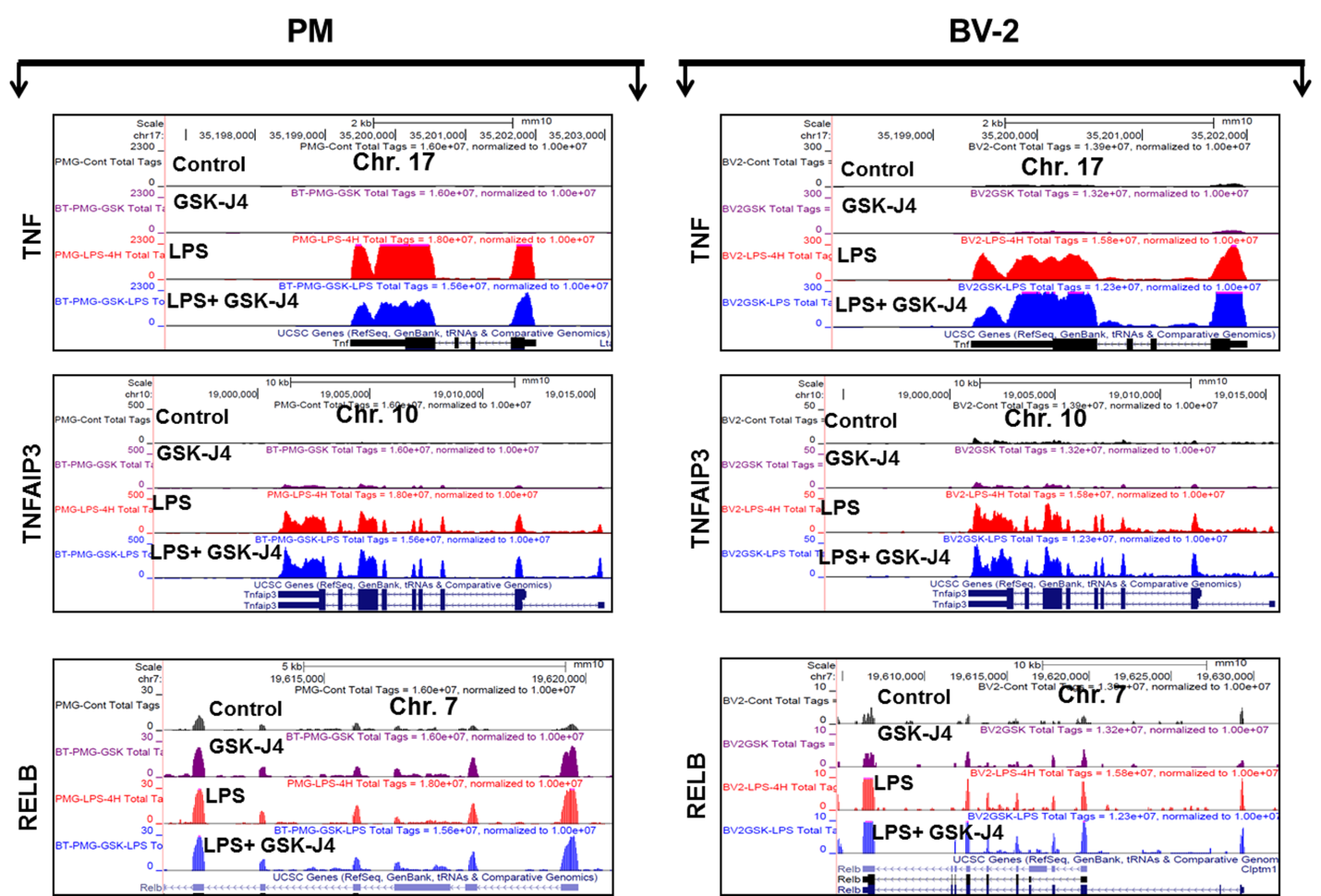
**

**Figure S3 GSK-J4** **did not affect a specific subset of LPS-inducible genes in PM and BV-2 microglial cells.** UCSC Browser images representing the normalized RNA-seq read densities of inflammatory genes that were un-affected by GSK-J4 after 4 h in LPS-induced PM and BV-2 microglial cells compared with the control.

**
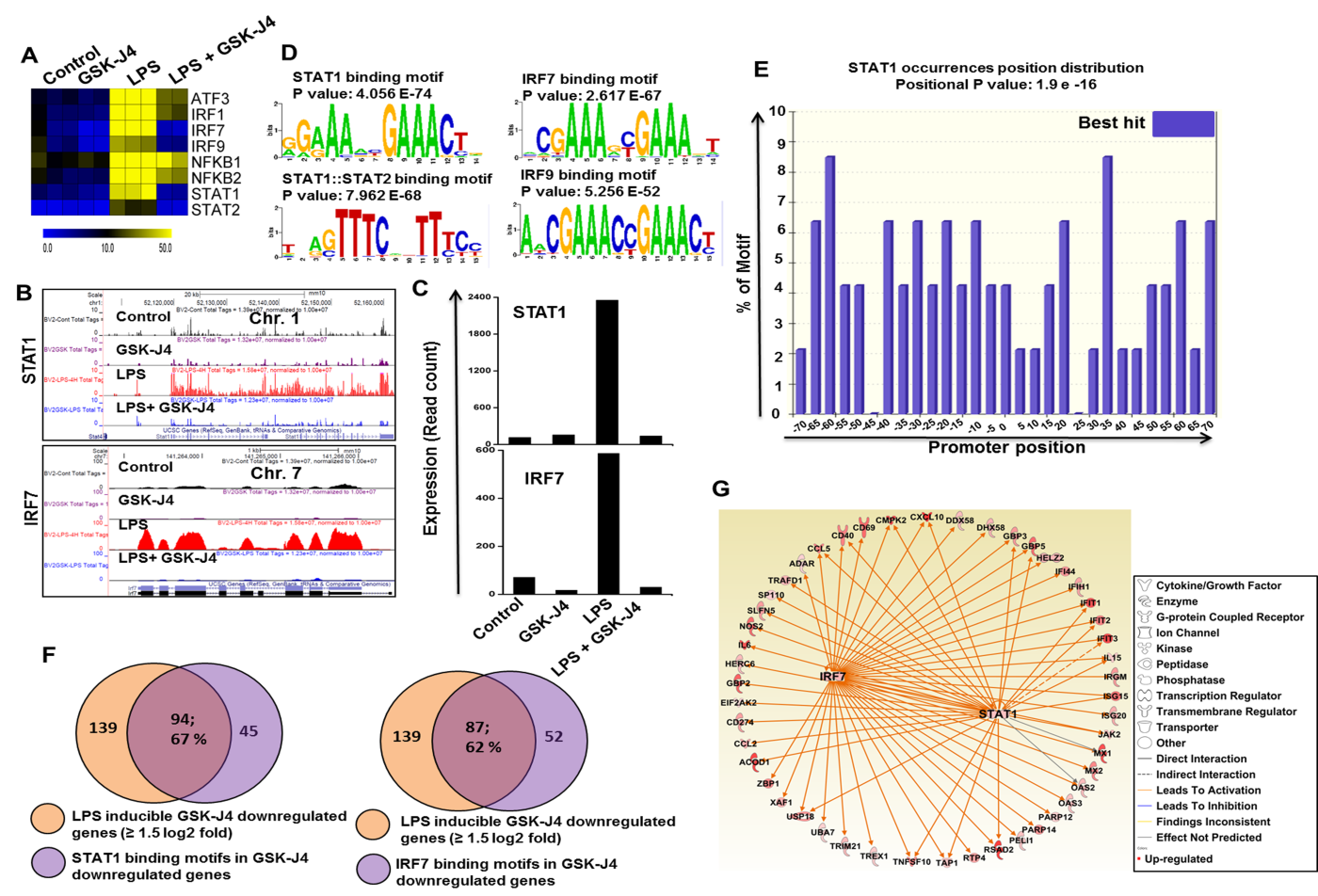
**

**Figure S4 Inhibitory effect of GSK-J4 on LPS-induced key TFs in BV-2 microglial cells.** (A) A heat map representation of TFs expression levels that were selectively down-regulated (*P* ≤ 0.01, and log_2-_fold change ≥ 1.5) by GSK-J4 at 4 h after LPS-stimulation, from three independent BV-2 microglial cells experiments. Heat maps were generated with the Multi Experiment Viewer (version 4.8) software. (B) UCSC Browser images representing the normalized RNA-seq read density in GSK-J4 down-regulated inflammatory genes at 4 h in the LPS-induced BV-2 microglial cells compared with controls. (C) Transcript abundance (in Read count) was evaluated using RNA-seq in GSK-J4 down-regulated TFs at 4 h in the LPS-induced BV-2 microglial cells. (D, E) Patterns of TF motif enrichment within the promoters of the GSK-J4 down-regulated genes (*P* ≤ 0.01, and log_2-_fold change ≥ 1.5) in LPS-induced BV-2 microglial cells. (F) Venn diagrams of GSK-J4 down-regulated genes associated with STAT1 and IRF7 in 4 h LPS treated BV-2 microglial cells. (G) The activity of highly connected positive regulators of the inflammatory genes STAT1 and IRF7 led to the activation of this network, as assessed using the IPA molecule activity predictor in GSK-J4 down-regulated genes in BV-2 microglial cells.

**
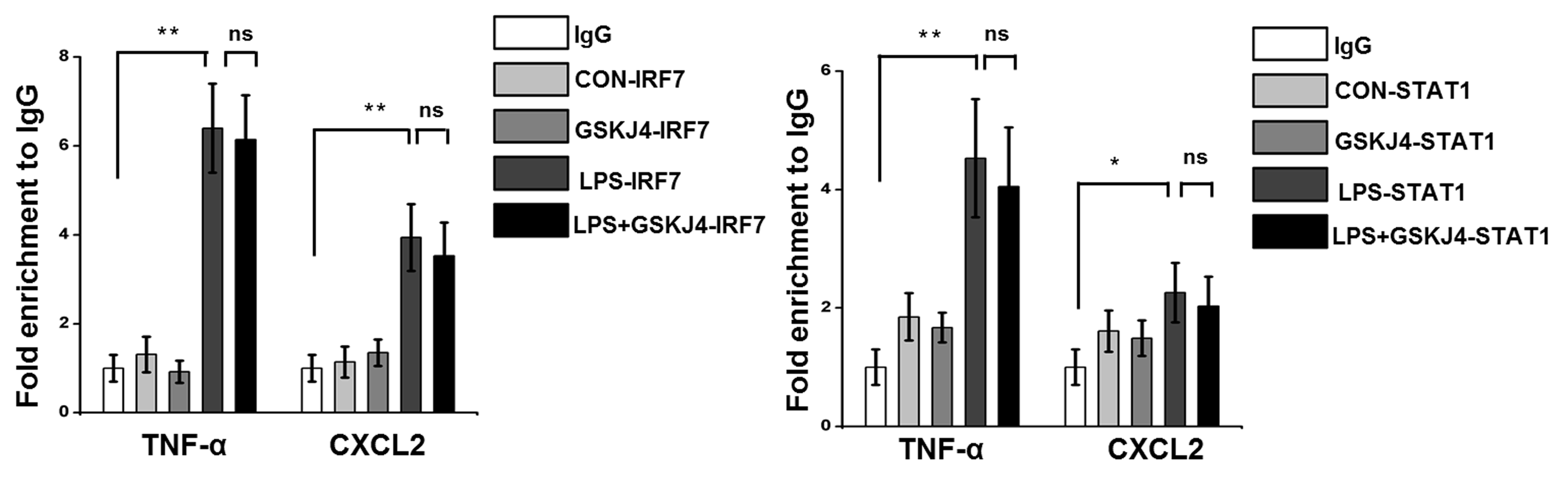
**

**Figure S5 GSK-J4 did not affect STAT1 and IRF7 binding at selected gene promoter regions.** ChIP assay was used to determine the presence of STAT1 and IRF7 at selected genes. The ChIP-enriched samples were analyzed using quantitative PCR with selected gene primers. STAT1 and IRF7 binding was increased following LPS exposure, though it did not reduce STAT1 and IRF7 binding at the promoters of the TNF-α and CXCL2 genes in the GSK-J4-treated BV-2 microglial cells. The graphs represent the mean fold values of enrichment relative to the IgG control from three independent experiments. **P* <0.01, ***P* <0.001 and ns is non-significant compared with the control.

**
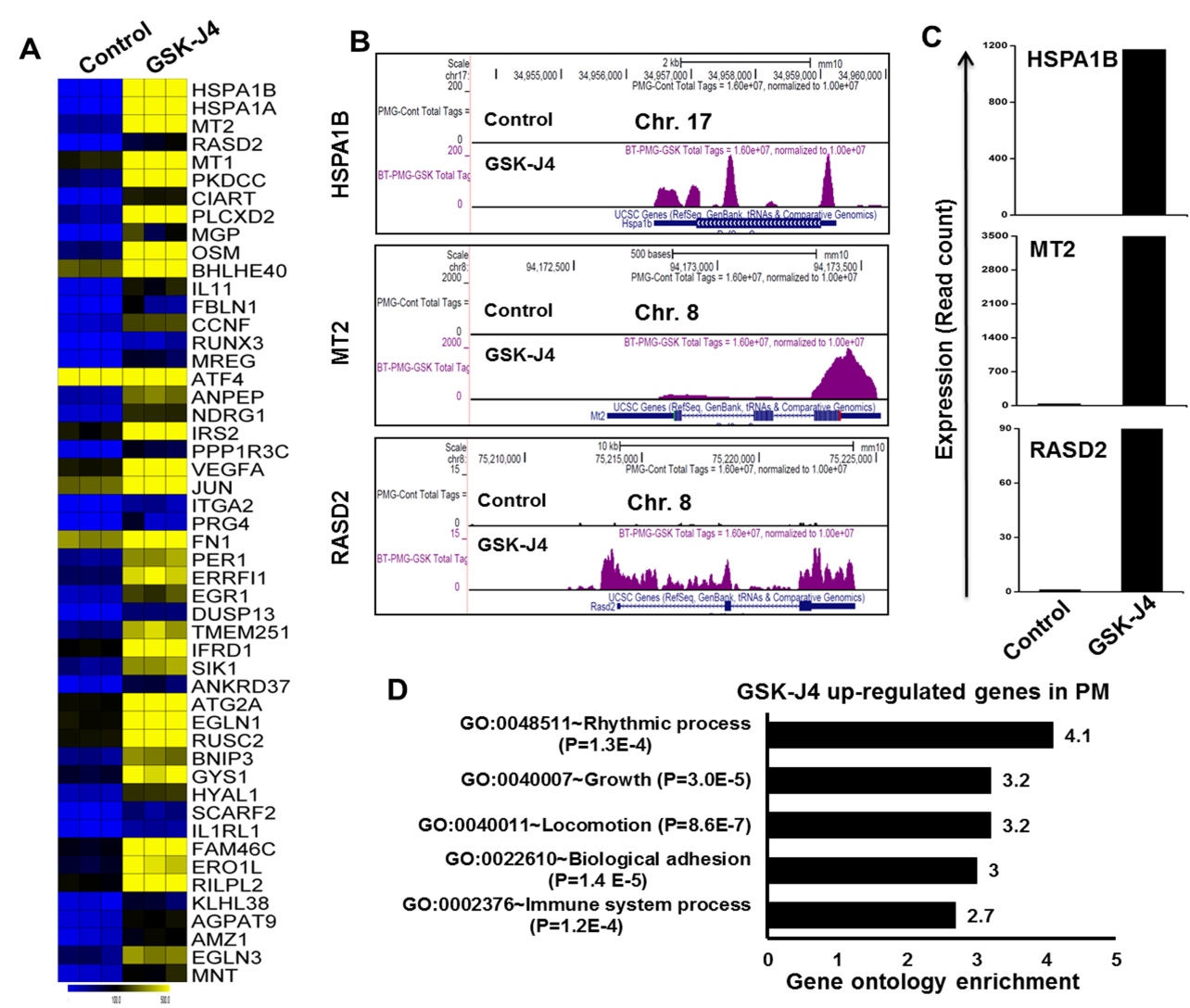
**

**Figure S6 Effect of GSK-J4 alone in PM.** (A) A heat map representing the top 50 transcripts in PM that were up-regulated by GSK-J4 alone in the RNA-seq gene expression data (*P* ≤ 0.01, and log_2_-fold change ≥ 1.5). PM cells are compared to the control. Each row shows the relative expression level for a single gene, and each column shows the expression level of a single sample. Heat maps were generated with the Multi Experiment Viewer (version 4.8) software. (B) UCSC Browser images representing normalized RNA-seq read densities in PM. (C) Transcript abundance (in Read count) was evaluated using RNA-seq in GSK-J4 alone treated. (D) Gene ontology analysis of the functional annotations that were associated with up-regulated genes at 4 h after GSK-J4 alone stimulation in the PM.

**Supporting Information Table 1:** STAT1, IRF7 binding motif transcript in GSK-J4 inhibited LPS-induced inflammatory genes in PM and BV-2 microglial cells (*P* ≤ 0.001, and log_2-_fold change ≥ 1.5)

| **PM** | | | | |  |
| --- | --- | --- | --- | --- | --- |
| **Top 150_STAT1 binding motif (score >=0.819)** | | | | | |
| **Gene symbol** | **Score** | **Position** | **Sequence** | | **Strand** |
| BST2 | 1 | -166 | GGAAAATGAAACTG | | + |
| SLFN9 | 1 | -48 | GGAAAACGAAACTG | | - |
| SLFN8 | 1 | -139 | GGAAAACGAAACTG | | - |
| RTP4 | 0.982463 | -26 | GGAAACTGAAACTG | | + |
| IFIT1BL2 | 0.982463 | -52 | GGAAAGTGAAACTG | | + |
| IFIT1 | 0.982463 | -107 | GGAAAGTGAAACTG | | - |
| FCGR4 | 0.980612 | -74 | GGAAAAGGAAACTG | | + |
| GM4951 | 0.972428 | -134 | GGAAAGTGAAACTA | | - |
| GBP3 | 0.97186 | -56 | AGAAACTGAAACTG | | - |
| IL27 | 0.97186 | -80 | AGAAAGTGAAACTG | | + |
| IFITM3 | 0.97186 | 18 | AGAAACCGAAACTG | | + |
| IRGM1 | 0.97186 | -99 | AGAAACCGAAACTG | | + |
| PHF11D | 0.971732 | -6 | GGGAAACGAAACTA | | + |
| ZBP1 | 0.96723 | -55 | AGAAAATGAAACTT | | - |
| DDX58 | 0.963875 | -1 | GGAAATCGAAACTG | | - |
| ISG20 | 0.961825 | -310 | AGAAACTGAAACTA | | + |
| CXCL10 | 0.960296 | -214 | GGAAAGTGAAACTT | | + |
| USP18 | 0.960296 | -86 | GGAAAGCGAAACTC | | - |
| IFIT3 | 0.960296 | -159 | GGAAAGTGAAACTT | | - |
| IFIT3B | 0.960296 | -191 | GGAAAGTGAAACTT | | - |
| TOR3A | 0.958093 | -471 | GGAAAATGAAACAG | | - |
| SLC7A11 | 0.958093 | -315 | GGAAAATGAAACAG | | - |
| EIF2AK2 | 0.958093 | 17 | GGAAAACGAAACAG | | + |
| ISG15 | 0.957293 | -79 | GGAAAAGGAAACCG | | + |
| IL1A | 0.954195 | -869 | GGGAACTGAAACTA | | + |
| IGTP | 0.953628 | -41 | AGGAACTGAAACTG | | + |
| IRGM2 | 0.953628 | -71 | AGGAACTGAAACTG | | + |
| TRIM34A | 0.949693 | 7 | AGAAACTGAAACTC | | - |
| TRIM30A | 0.94843 | -39 | AGAAAAAGAAACTA | | - |
| TRIM30D | 0.94843 | -44 | AGAAAAAGAAACTA | | - |
| BC147527 | 0.94843 | -111 | AGAAAAAGAAACTA | | + |
| PHF11B | 0.94843 | -51 | AGAAAAAGAAACTA | | + |
| PHF11A | 0.94843 | -30 | AGAAAAAGAAACTA | | + |
| CD274 | 0.945954 | -178 | AAAAAACGAAACTA | | - |
| OAS1C | 0.944487 | -111 | GGAAATGGAAACTG | | - |
| OAS1B | 0.944487 | -26 | GGAAATGGAAACTG | | + |
| OAS1A | 0.944487 | -21 | GGAAATGGAAACTG | | + |
| OAS1G | 0.944487 | -19 | GGAAATGGAAACTG | | + |
| H2-T24 | 0.944203 | -370 | CGAAACCGAAACTG | | - |
| IFIT1BL1 | 0.943911 | -107 | AGAAAATGAAACCT | | - |
| PNPT1 | 0.943507 | -75 | CGGAAACGAAACTG | | + |
| GM12250 | 0.943094 | -43 | AGAACATGAAACTG | | - |
| OAS3 | 0.943094 | -76 | AGAAAACGAAAGTG | | + |
| MLKL | 0.943094 | -296 | AGAAAACGAAAGTG | | - |
| APOBEC3 | 0.942437 | -143 | AGAAAGGGAAACTA | | - |
| OAS2 | 0.941707 | -79 | GGAAATCGAAACTC | | - |
| CD40 | 0.940929 | -501 | AGAAAGAGAAACTG | | + |
| BRIP1 | 0.938705 | -62 | GGAAAAGGAAACAG | | - |
| SPPL2A | 0.936977 | -9 | GGAAACTGAAACCC | | + |
| PVRL4 | 0.936655 | -106 | AGAAAAGGAAACCA | | - |
| TRIM21 | 0.936161 | 12 | GGAAACTGAAAGTG | | + |
| TGTP1 | 0.935039 | -84 | AGGAATCGAAACTG | | + |
| H2-T10 | 0.934167 | -218 | CGAAAGTGAAACTA | | - |
| GBP7 | 0.930823 | -50 | GAGAACTGAAACTG | | - |
| SERTAD3 | 0.930379 | -664 | GGAAAACGAAAGCG | | + |
| NCOA7 | 0.929365 | 6 | GGAAACAGAAACTC | | + |
| SOCS1 | 0.928214 | -84 | GGAAAGAGAAACCG | | + |
| IFI44 | 0.926888 | -9 | GAAAACTGAAACTC | | - |
| FAM26F | 0.926374 | -65 | AGAAAGTGAAACCC | | + |
| GM12185 | 0.926374 | -76 | AGAAAGTGAAACCT | | + |
| TRIM26 | 0.923706 | -157 | AGAAAAGGAAAGTG | | - |
| RBM43 | 0.922341 | -375 | AGAAATAGAAACTG | | - |
| PSTPIP2 | 0.920927 | -853 | AGAAAATGAAAGTT | | - |
| LGALS3BP | 0.920431 | -45 | GAAAATCGAAACTA | | + |
| SP110 | 0.920289 | 6 | GAAAAATGAAAGTG | | - |
| TOR1AIP2 | 0.920189 | -325 | CGGAAATGAAACCG | | + |
| RHOH | 0.919222 | -244 | AGGAAATGAAACAA | | - |
| IFI47 | 0.918936 | -459 | GAGAAAGGAAACTA | | + |
| GPR18 | 0.918745 | -723 | GGGAAGTGAAACCC | | - |
| LGALS9 | 0.918178 | 12 | GGAAACAGAAACCA | | - |
| FPR2 | 0.915701 | -36 | GAAAACTGAAACCA | | - |
| XAF1 | 0.915589 | 8 | AAGAAACGAAACTC | | + |
| SERPINB9 | 0.915522 | -49 | AGAAAGTGAAAGTA | | - |
| SLC2A6 | 0.914082 | -649 | AAAAAACGAAACAG | | - |
| TGTP2 | 0.911721 | -77 | AGGAATCGAAACCG | | + |
| CLCA3A1 | 0.911716 | -23 | AGAAATGGAAACTT | | - |
| MMP25 | 0.911132 | 26 | GGGAACAGAAACTT | | + |
| PARP10 | 0.910795 | -1 | CAAAAGTGAAACTG | | - |
| SP100 | 0.910254 | -50 | GAAAAACGAAAGTA | | - |
| CASP8 | 0.910099 | -111 | CAGAAACGAAACTG | | + |
| HELZ2 | 0.910099 | 15 | CAGAAACGAAACTG | | + |
| GYPC | 0.909625 | -93 | GGAAACAGAAACAG | | + |
| CCL12 | 0.908333 | -296 | AAGAAAGGAAACTA | | - |
| TRIM14 | 0.907786 | -5 | AGAAATCGAAACCT | | - |
| ZFP263 | 0.905934 | -689 | AGAAAAGGAAACAC | | - |
| KLRK1 | 0.905519 | -104 | AGAAGATGAAACTG | | + |
| LRCH1 | 0.905013 | -166 | CAAAAACGAAACCG | | + |
| TSPO | 0.904826 | -440 | GGAAAACAAAACTA | | - |
| GM5431 | 0.904676 | -26 | GGGAATAGAAACTA | | + |
| PSME1 | 0.902806 | 6 | GGAAAGCGAAAGCA | | - |
| CCL8 | 0.902111 | -82 | GGGAAATGAAAGCA | | - |
| MX2 | 0.902057 | -946 | GAGAAATGAAAGTG | | + |
| PARP14 | 0.901543 | -61 | AGGAAATGAAAGCG | | + |
| TNFSF10 | 0.90076 | -56 | CAAAAGTGAAACTA | | - |
| UBA7 | 0.90076 | -30 | CAAAAGTGAAACTA | | - |
| SLFN4 | 0.90076 | -228 | CAAAAGTGAAACTA | | - |
| MB21D1 | 0.900173 | -457 | AGAAATAGAAACTC | | - |
| DBNL | 0.900064 | -710 | CAGAAATGAAACTA | | - |
| CD80 | 0.897486 | 27 | AAAAAGAGAAACTA | | - |
| ZUFSP | 0.897325 | -144 | GGAAACTGCAACTG | | + |
| CLEC4D | 0.896733 | -3 | GGAAAAGGAAATTG | | - |
| GBP2 | 0.896592 | -62 | TGAAAGTGAAACTA | | - |
| OASL1 | 0.895957 | -652 | GAAAAGAGAAACTC | | - |
| RSAD2 | 0.895957 | -109 | GAAAACAGAAACTC | | + |
| TIMD4 | 0.895761 | -73 | GGGAAGTGAAAGTT | | + |
| CCL5 | 0.895474 | -153 | GGAAAAGAAAACTG | | - |
| XRN1 | 0.895443 | -260 | AGAAAGAGAAACCT | | - |
| GBP10 | 0.895194 | -296 | GGAAAGAGAAAGTA | | + |
| BC094916 | 0.894253 | -80 | GGAAATCGAAAGCG | | + |
| OGFR | 0.894253 | 28 | GGAAAGTGAAAGAG | | - |
| TNFSF15 | 0.893558 | -901 | GGGAAATGAAAGAG | | + |
| ARRDC4 | 0.892694 | -485 | GGAAAATGGAACTC | | - |
| SAMSN1 | 0.892694 | -256 | GGAAAACAAAACTT | | - |
| DUSP28 | 0.892207 | -860 | CAAAATTGAAACTG | | + |
| ATP10A | 0.892021 | -127 | GAGAAACGAAAGTA | | + |
| TNFRSF1B | 0.891915 | -607 | AAAAAATGAAACAT | | + |
| LCP2 | 0.891093 | -86 | GGTAAAGGAAACTA | | + |
| CAR13 | 0.890675 | -45 | GGAACCCGAAACCC | | - |
| FAM46A | 0.890067 | -729 | GAGAATCGAAACTC | | + |
| IFIH1 | 0.889953 | 31 | CGAAACAGAAACCG | | + |
| MX1 | 0.888916 | -69 | GAGAATTGAAACCG | | - |
| C2 | 0.888737 | -274 | AGGACTTGAAACTG | | - |
| XKR8 | 0.887519 | -671 | AAAACATGAAACTT | | - |
| GSDMD | 0.887477 | -13 | CAAAAGTGAAACCG | | - |
| IFI204 | 0.885814 | -63 | AAGAAATGAAACAA | | + |
| GBP2B | 0.88519 | -824 | GGAAAAAGAAAATG | | + |
| ABCC1 | 0.884871 | -337 | AGATAAGGAAACTG | | - |
| CXCL16 | 0.884574 | 35 | GGGAAGTGAAAGCA | | + |
| CLEC2D | 0.884285 | -185 | GAGAAACGAAACAC | | + |
| MYD88 | 0.884218 | -19 | GGAAAGCGAAAGAA | | + |
| TMEM106A | 0.884002 | -803 | AGAAAGGGAAAGTT | | + |
| INTU | 0.882767 | -699 | GGAAGATGAAACCA | | + |
| CCL2 | 0.882155 | -174 | GAAGAGAGAAACTG | | - |
| IFI202B | 0.88094 | 21 | AGAAAATGACACCG | | - |
| GPD2 | 0.880585 | -694 | GAAAACCGAAAGTC | | + |
| PSMB9 | 0.880072 | -378 | AGAAACCGAAAGCC | | + |
| TRIM12C | 0.879889 | 4 | GAGAAACGAAAGTT | | - |
| SLC15A3 | 0.879889 | -107 | GAGAAATGAAAGTC | | - |
| TDRD7 | 0.879864 | -821 | CAAAACAGAAACTG | | - |
| H2-T23 | 0.879197 | -295 | GGAAGGGGAAACTG | | + |
| IL15 | 0.879093 | -601 | GGGAAGCCAAACTG | | + |
| H2-M2 | 0.878664 | -170 | AAGAAGGGAAACTC | | - |
| GBP5 | 0.878003 | -168 | TGAAATTGAAACTA | | - |
| IL6 | 0.877241 | -283 | GGGAAAGAAAACTG | | - |
| GM1966 | 0.876687 | -696 | AGACAGTGAAACTA | | - |
| CCL7 | 0.875158 | -126 | AGGAATGGAAAATG | | - |
| PLEKHA2 | 0.875158 | -877 | GGAAACCGAGACTC | | - |
| ADGRE1 | 0.874865 | -226 | GGAAAGGGAAAGAG | | + |
| FNBP1L | 0.874007 | -687 | GGAAAGCGAACCCG | | - |
| CEPT1 | 0.875158 | -483 | GGACAGTGAAACTC | | - |
|  |  |  |  | |  |
| **Top 150_IRF7 binding motif (score >=0.8023)** | | | | | |
| **Gene symbol** | **Score** | **Position** | **Sequence** | | **Strand** |
| H2-T10 | 0.973222 | -217 | TCGAAAGTGAAACT | | - |
| H2-T24 | 0.958985 | -369 | TCGAAACCGAAACT | | - |
| MLKL | 0.943985 | -295 | AAGAAAACGAAAGT | | - |
| USP18 | 0.939209 | -85 | AGGAAAGCGAAACT | | - |
| IL27 | 0.93915 | -81 | CAGAAAGTGAAACT | | + |
| PSTPIP2 | 0.93527 | -852 | AAGAAAATGAAAGT | | - |
| GBP2 | 0.93129 | -504 | CTGAAAGTGAAAGT | | - |
| GM4951 | 0.930494 | -133 | AGGAAAGTGAAACT | | - |
| IFIT3 | 0.930494 | -158 | AGGAAAGTGAAACT | | - |
| IFIT3B | 0.930494 | -190 | AGGAAAGTGAAACT | | - |
| MX2 | 0.929133 | -941 | ATGAAAGTGAAACT | | + |
| TGTP2 | 0.92658 | -72 | TCGAAACCGAAACC | | + |
| IFI47 | 0.925265 | -36 | ACAAAAGTGAAACT | | - |
| SLFN4 | 0.925265 | -227 | ACAAAAGTGAAACT | | - |
| PARP10 | 0.925265 | 0 | ACAAAAGTGAAACT | | - |
| TNFSF10 | 0.922431 | -55 | CCAAAAGTGAAACT | | - |
| UBA7 | 0.922431 | -29 | CCAAAAGTGAAACT | | - |
| GBP2B | 0.920142 | -139 | AAGAAAGTGAAATT | | - |
| SCARF1 | 0.919707 | -123 | ACGAAAGGGAAAAT | | - |
| RSAD2 | 0.919583 | -122 | CTGAAAATGAAAGT | | - |
| TRIM34A | 0.919033 | 8 | AAGAAACTGAAACT | | - |
| BST2 | 0.918787 | -167 | AGGAAAATGAAACT | | + |
| TGTP1 | 0.917865 | -79 | TCGAAACTGAAACC | | + |
| CXCL10 | 0.917424 | -215 | TGGAAAGTGAAACT | | + |
| IFIT1BL2 | 0.917424 | -53 | TGGAAAGTGAAACT | | + |
| IFIT1 | 0.917424 | -106 | TGGAAAGTGAAACT | | - |
| ISG20 | 0.916198 | -311 | CAGAAACTGAAACT | | + |
| SERPINB9 | 0.915448 | -48 | GAGAAAGTGAAAGT | | - |
| IRGM1 | 0.914678 | -100 | TAGAAACCGAAACT | | + |
| MAGOHB | 0.913726 | -788 | TCGAAAGTAAAAGT | | + |
| TRIM21 | 0.912534 | 11 | AGGAAACTGAAAGT | | + |
| OAS3 | 0.912456 | -77 | GAGAAAACGAAAGT | | + |
| PARP14 | 0.911278 | -56 | ATGAAAGCGAAAGA | | + |
| FAM208B | 0.910909 | 1 | CAGAAAGTGAAAAC | | - |
| MYD88 | 0.909805 | -20 | CGGAAAGCGAAAGA | | + |
| FAM26F | 0.909579 | -66 | AAGAAAGTGAAACC | | + |
| GBP4 | 0.907306 | -82 | ACAAAACTGAAAGT | | - |
| GBP9 | 0.907306 | -102 | ACAAAACTGAAAGT | | - |
| GBP6 | 0.907306 | -152 | ACAAAACTGAAAGT | | - |
| GBP11 | 0.907306 | -115 | ACAAAACTGAAAGT | | - |
| TRIM56 | 0.907306 | -6 | ACAAAACTGAAAGT | | - |
| XAF1 | 0.907306 | -19 | ACAAAACTGAAAGT | | - |
| GBP10 | 0.906478 | -144 | ACAAAACTGAAAAT | | - |
| GBP3 | 0.905963 | -55 | TAGAAACTGAAACT | | - |
| OGFR | 0.903925 | 29 | AGGAAAGTGAAAGA | | - |
| CD274 | 0.903844 | -340 | ACGAAACTAAAAGT | | - |
| GBP7 | 0.903346 | -55 | CTGAAACTGAAACT | | - |
| RTP4 | 0.903187 | -76 | TGGAAACCGAAACT | | + |
| TNFRSF1B | 0.902607 | -12 | CTGAAAGCGAAACC | | - |
| ISG15 | 0.902317 | -68 | CCGAAACAGAAAAT | | + |
| SLC2A6 | 0.900988 | -654 | ACGAAACAGAAACT | | - |
| PSMB9 | 0.900335 | -379 | AAGAAACCGAAAGC | | + |
| ZBP1 | 0.900109 | -96 | TCGAAATCGAAATT | | - |
| OAS1C | 0.899464 | -116 | TGGAAACTGAAAGT | | - |
| OAS1B | 0.899464 | -21 | TGGAAACTGAAAGT | | + |
| RBM43 | 0.898726 | 1 | TGGAAAGCGAAAGC | | + |
| BID | 0.898696 | -564 | ACAAAAGTGAAAGA | | - |
| OAS1A | 0.898636 | -16 | TGGAAACTGAAAAT | | + |
| OAS1G | 0.898636 | -14 | TGGAAACTGAAAAT | | + |
| PSD4 | 0.897571 | -266 | ATGAAAGTGAAACA | | - |
| GM4841 | 0.897571 | -162 | ATGAAAGTGAAACA | | - |
| IIGP1 | 0.897571 | -159 | ATGAAAGTGAAACA | | - |
| SLAMF8 | 0.897365 | -17 | TTGAAAGCGAAAGC | | - |
| SERTAD3 | 0.897254 | -665 | CGGAAAACGAAAGC | | + |
| DUSP28 | 0.896727 | -894 | ATGAAAGTGAAACC | | + |
| GM12185 | 0.896509 | -77 | TAGAAAGTGAAACC | | + |
| IFITM3 | 0.896219 | 17 | GAGAAACCGAAACT | | + |
| SLFN9 | 0.895973 | -47 | GGGAAAACGAAACT | | - |
| SLFN8 | 0.895973 | -138 | GGGAAAACGAAACT | | - |
| IFIT1BL1 | 0.895037 | -106 | CAGAAAATGAAACC | | - |
| SLC25A22 | 0.894322 | -868 | CCCAAAATGAAAGA | | + |
| GSDMD | 0.89286 | -12 | ACAAAAGTGAAACC | | - |
| PNPT1 | 0.891973 | -76 | ACGGAAACGAAACT | | + |
| LRCH1 | 0.889868 | -167 | ACAAAAACGAAACC | | + |
| TBC1D13 | 0.88981 | -688 | TAGAAAATGAAAAA | | - |
| TOR3A | 0.887225 | -470 | AGGAAAATGAAACA | | - |
| IFI205 | 0.883058 | 21 | GAGAAAGTGAAAAA | | - |
| GBP5 | 0.88277 | -161 | TCTAAACTGAAATT | | - |
| STARD3 | 0.881805 | -278 | TCAAAAACGAAAAA | | + |
| PSME1 | 0.880267 | 7 | GGGAAAGCGAAAGC | | - |
| CD40 | 0.879632 | -502 | AAGAAAGAGAAACT | | + |
| LGALS3BP | 0.879467 | -897 | ACAAAACCGAAACA | | - |
| DDX58 | 0.879224 | 0 | AGGAAATCGAAACT | | - |
| OAS2 | 0.879224 | -78 | AGGAAATCGAAACT | | - |
| RNF31 | 0.878906 | -855 | GTGAAAGCGAAAGC | | - |
| TAP2 | 0.878906 | 20 | GTGAAAGCGAAAGC | | + |
| TAP1 | 0.878906 | -563 | GTGAAAGCGAAAGC | | + |
| GM12250 | 0.878768 | -48 | ATGAAACTGAAAGC | | - |
| CASP4 | 0.878122 | 0 | AGTAAAATGAAAGT | | - |
| IFI44L | 0.877293 | -194 | AGTAAAATGAAAAT | | - |
| TRIM26 | 0.876245 | -162 | AGGAAAGTGAAATC | | - |
| IGTP | 0.875933 | -36 | CTGAAACTGAAAGC | | + |
| IRGM2 | 0.875933 | -66 | CTGAAACTGAAAGC | | + |
| NOS2 | 0.875728 | -885 | ATGAAAGTGAAATA | | - |
| SPPL2A | 0.875136 | -10 | AGGAAACTGAAACC | | + |
| DHX58 | 0.874619 | -6 | ATGAAACTGAAACA | | - |
| SLC7A11 | 0.874155 | -314 | TGGAAAATGAAACA | | - |
| TMEM106A | 0.873393 | -804 | CAGAAAGGGAAAGT | | + |
| TMEM2 | 0.871914 | -361 | TCGAAACTGAACCT | | - |
| SNX10 | 0.871861 | -529 | ACAAAAGTGAAATA | | + |
| PRDX1 | 0.8718 | -614 | CAGAAAATGAAAAG | | - |
| SP100 | 0.871467 | -49 | AGAAAAACGAAAGT | | - |
| TNFSF15 | 0.870944 | -896 | ATGAAAGAGAAAAT | | + |
| CXCL9 | 0.870758 | -867 | ACAAAAGTGAATGT | | - |
| IRG1 | 0.870451 | -67 | ACAAAAGTGAAAGG | | + |
| SOCS1 | 0.868806 | -79 | GAGAAACCGAAAGC | | + |
| BC147527 | 0.867925 | -112 | AAGAAAAAGAAACT | | + |
| DTX3L | 0.866657 | -845 | CCAAAAGCAAAACT | | + |
| DDX24 | 0.865816 | -941 | ATGAAAACAAAAAT | | - |
| EIF2AK2 | 0.864411 | 16 | GGGAAAACGAAACA | | + |
| EPSTI1 | 0.863175 | -314 | TGGAAAGTGAAATC | | - |
| TSPO | 0.863013 | -439 | AGGAAAACAAAACT | | - |
| CXCL11 | 0.862913 | -123 | ACAAAAGAGAAACT | | + |
| OASL1 | 0.862913 | -159 | ACAAAAGAGAAACT | | + |
| SP110 | 0.862752 | 7 | AGAAAAATGAAAGT | | - |
| MITD1 | 0.861348 | -239 | TCGAAACAGAAAGA | | + |
| MORC3 | 0.860736 | -640 | AACAAAACGAAAAA | | - |
| SIGLECE | 0.86059 | -853 | ACTAAAATGCAAGT | | - |
| TNFRSF14 | 0.859327 | -66 | GTGAAAATGAAAGA | | - |
| ATP10A | 0.859019 | -533 | TAGAAAAAGAAAAT | | + |
| AZI2 | 0.858661 | 0 | ACGAAACCGGAAGC | | - |
| CCL22 | 0.857333 | -897 | CAGAAAGTGGAAAT | | + |
| KLRK1 | 0.856883 | -540 | TAGAAAATAAAAAT | | - |
| TMA16 | 0.856198 | -516 | ACAAAAAAGAAAGT | | + |
| GM1966 | 0.855729 | -77 | AACAAAACGAAACC | | + |
| TPM4 | 0.85553 | -945 | CAGAAAGTGAGAAT | | + |
| IFIH1 | 0.855512 | 30 | TCGAAACAGAAACC | | + |
| TRIM14 | 0.855474 | -4 | CAGAAATCGAAACC | | - |
| ADAR | 0.855095 | -808 | CTGAAAATAAAAGT | | + |
| PSMA5 | 0.854602 | -302 | AAGAAATTGAAAAA | | + |
| CCL5 | 0.853544 | -132 | CATAAAATGAAAAC | | - |
| BC094916 | 0.851811 | -81 | AGGAAATCGAAAGC | | + |
| PYDC3 | 0.851811 | -75 | AGGAAATCGAAAGC | | + |
| TOR1AIP2 | 0.851396 | -465 | AAAAAAACGAAAGA | | + |
| MGAT4A | 0.851054 | -29 | ACGAAAGGAAAACT | | - |
| N4BP1 | 0.850852 | -465 | TATAAAGTGAAACC | | - |
| TMEM243 | 0.850845 | -928 | ATGAAACTGAATAT | | + |
| GM5431 | 0.850228 | -399 | CAGAAAGAGAAAGA | | + |
| AIM1 | 0.850074 | -59 | TGGAAAATGAAAAG | | - |
| SAMSN1 | 0.849943 | -255 | TGGAAAACAAAACT | | - |
| TRIM34B | 0.848921 | -434 | ATTAAAACGAAACA | | - |
| IL13RA1 | 0.848915 | -648 | TCGAAACCTAAAAC | | + |
| IFI35 | 0.848144 | 16 | ATGAAAGTGGAAGT | | + |
| DRAM1 | 0.847637 | -182 | GCAAAAGCGAAAGG | | + |
| CLEC5A | 0.845716 | -376 | GCCAAAGAGAAAAT | | + |
| ACPP | 0.845316 | -213 | CGGAAAGCAAAAGA | | + |
|  |  |  |  | |  |
| **BV-2 microglial cells** | | | | | |
| **STAT1 binding motif (score >=0.840)** | | | | | |
| **Gene symbol** | **Score** | **Position** | **Sequence** | **Strand** | |
| SLFN8 | 1 | -139 | GGAAAACGAAACTG | - | |
| RTP4 | 0.982463 | -26 | GGAAACTGAAACTG | + | |
| IFIT1BL2 | 0.982463 | -52 | GGAAAGTGAAACTG | + | |
| IFIT1 | 0.982463 | -107 | GGAAAGTGAAACTG | - | |
| GBP3 | 0.97186 | -56 | AGAAACTGAAACTG | - | |
| IRGM1 | 0.97186 | -99 | AGAAACCGAAACTG | + | |
| PHF11D | 0.971732 | -6 | GGGAAACGAAACTA | + | |
| ZBP1 | 0.96723 | -55 | AGAAAATGAAACTT | - | |
| DDX58 | 0.963875 | -1 | GGAAATCGAAACTG | - | |
| ISG20 | 0.961825 | -310 | AGAAACTGAAACTA | + | |
| CXCL10 | 0.960296 | -214 | GGAAAGTGAAACTT | + | |
| USP18 | 0.960296 | -86 | GGAAAGCGAAACTC | - | |
| IFIT3 | 0.960296 | -159 | GGAAAGTGAAACTT | - | |
| IFIT3B | 0.960296 | -191 | GGAAAGTGAAACTT | - | |
| TOR3A | 0.958093 | -471 | GGAAAATGAAACAG | - | |
| EIF2AK2 | 0.958093 | 17 | GGAAAACGAAACAG | + | |
| ISG15 | 0.957293 | -79 | GGAAAAGGAAACCG | + | |
| IL1A | 0.954195 | -869 | GGGAACTGAAACTA | + | |
| IGTP | 0.953628 | -41 | AGGAACTGAAACTG | + | |
| IRGM2 | 0.953628 | -71 | AGGAACTGAAACTG | + | |
| TARM1 | 0.951532 | -340 | GGAAAGAGAAACTG | + | |
| TRIM34A | 0.949693 | 7 | AGAAACTGAAACTC | - | |
| TRIM30A | 0.94843 | -39 | AGAAAAAGAAACTA | - | |
| TRIM30D | 0.94843 | -44 | AGAAAAAGAAACTA | - | |
| PHF11B | 0.94843 | -51 | AGAAAAAGAAACTA | + | |
| PHF11A | 0.94843 | -30 | AGAAAAAGAAACTA | + | |
| CD274 | 0.945954 | -178 | AAAAAACGAAACTA | - | |
| OAS1B | 0.944487 | -26 | GGAAATGGAAACTG | + | |
| APOL9A | 0.944487 | -56 | GGAAATGGAAACTG | - | |
| APOL9B | 0.944487 | -83 | GGAAATGGAAACTG | - | |
| H2-T24 | 0.944203 | -370 | CGAAACCGAAACTG | - | |
| IFIT1BL1 | 0.943911 | -107 | AGAAAATGAAACCT | - | |
| GM12250 | 0.943094 | -43 | AGAACATGAAACTG | - | |
| OAS3 | 0.943094 | -76 | AGAAAACGAAAGTG | + | |
| OAS2 | 0.941707 | -79 | GGAAATCGAAACTC | - | |
| CD40 | 0.940929 | -501 | AGAAAGAGAAACTG | + | |
| TRIM21 | 0.936161 | 12 | GGAAACTGAAAGTG | + | |
| TRIM25 | 0.935039 | -7 | AGGAATCGAAACTG | - | |
| GBP7 | 0.930823 | -50 | GAGAACTGAAACTG | - | |
| IFI44 | 0.926888 | -9 | GAAAACTGAAACTC | - | |
| SP110 | 0.920289 | 6 | GAAAAATGAAAGTG | - | |
| IFI47 | 0.918936 | -459 | GAGAAAGGAAACTA | + | |
| LGALS9 | 0.918178 | 12 | GGAAACAGAAACCA | - | |
| XAF1 | 0.915589 | 8 | AAGAAACGAAACTC | + | |
| SERPINB9 | 0.915522 | -49 | AGAAAGTGAAAGTA | - | |
| OSBP2 | 0.912288 | -499 | GGGAATTGAAACCA | + | |
| PARP10 | 0.910795 | -1 | CAAAAGTGAAACTG | - | |
| SP100 | 0.910254 | -50 | GAAAAACGAAAGTA | - | |
| HELZ2 | 0.910099 | 15 | CAGAAACGAAACTG | + | |
| CCL12 | 0.908333 | -296 | AAGAAAGGAAACTA | - | |
| TRIM14 | 0.907786 | -5 | AGAAATCGAAACCT | - | |
| GM5431 | 0.904676 | -26 | GGGAATAGAAACTA | + | |
| MX2 | 0.902057 | -946 | GAGAAATGAAAGTG | + | |
| PARP14 | 0.901543 | -61 | AGGAAATGAAAGCG | + | |
| TNFSF10 | 0.90076 | -56 | CAAAAGTGAAACTA | - | |
| UBA7 | 0.90076 | -30 | CAAAAGTGAAACTA | - | |
| SLFN4 | 0.90076 | -228 | CAAAAGTGAAACTA | - | |
| ZUFSP | 0.897325 | -144 | GGAAACTGCAACTG | + | |
| GBP2 | 0.896592 | -62 | TGAAAGTGAAACTA | - | |
| OLR1 | 0.896545 | 22 | AAAAACTGAAACAG | - | |
| OASL1 | 0.895957 | -652 | GAAAAGAGAAACTC | - | |
| RSAD2 | 0.895957 | -109 | GAAAACAGAAACTC | + | |
| CCL5 | 0.895474 | -153 | GGAAAAGAAAACTG | - | |
| FAM46A | 0.890067 | -729 | GAGAATCGAAACTC | + | |
| IFIH1 | 0.889953 | 31 | CGAAACAGAAACCG | + | |
| MX1 | 0.888916 | -69 | GAGAATTGAAACCG | - | |
| IFI204 | 0.885814 | -63 | AAGAAATGAAACAA | + | |
| CLEC2D | 0.884285 | -185 | GAGAAACGAAACAC | + | |
| TRIM12C | 0.879889 | 4 | GAGAAACGAAAGTT | - | |
| IL15 | 0.879093 | -601 | GGGAAGCCAAACTG | + | |
| GBP5 | 0.878003 | -168 | TGAAATTGAAACTA | - | |
| IL6 | 0.877241 | -283 | GGGAAAGAAAACTG | - | |
| SAMD9L | 0.873974 | 24 | CAGAATCGAAACTG | + | |
| DHX58 | 0.872524 | -1 | TAGAAATGAAACTG | - | |
| MNDA | 0.872086 | -38 | GGAAATTGAAAGCT | + | |
| MNDAL | 0.872086 | -37 | GGAAATCGAAAGCT | + | |
| IFI205 | 0.872086 | -135 | GGAAATTGAAAGCT | + | |
| PYHIN1 | 0.871763 | -439 | AGAAAAGGAAAGAA | - | |
| PPM1K | 0.870395 | -341 | CAGAAGTGAAACTC | + | |
| ADAR | 0.868703 | -738 | GAGAAATGAAAGCA | + | |
| TREX1 | 0.86849 | -7 | AGGAAGTAAAACTG | - | |
| SRC | 0.867334 | -846 | AGACAGGGAAACTG | - | |
| EPSTI1 | 0.865231 | -315 | GGAAAGTGAAATCA | - | |
| TRIM12A | 0.864024 | 13 | TGGAAATGAAACAG | - | |
| TRIM56 | 0.863222 | -1 | GAGAAACAAAACTG | - | |
| IL1RN | 0.862035 | -75 | AAAAAGAGAAACCT | - | |
| MMP13 | 0.860608 | -149 | GGAAATGGAAAATG | - | |
| CD69 | 0.859743 | -638 | GGAAACAGAAAGCT | - | |
| AI607873 | 0.857829 | -42 | GGAAATCGAAAATT | + | |
| MS4A6C | 0.8481 | -497 | GAAAAATCAAACCA | - | |
| SLC7A2 | 0.845166 | -576 | AGAAACGGATACTT | - | |
| SP140 | 0.843194 | -278 | CAAAAATGATACTG | + | |
| CCL2 | 0.842986 | -174 | GAAGAGAGAAACTG | - | |
| CCL7 | 0.841464 | -126 | AGGAATGGAAAATG | - | |
|  |  |  |  |  | |
| **IRF7 binding motif (score >=0.8023)** | | | | | |
| **Gene symbol** | **Score** | **Position** | **Sequence** | | **Strand** |
| H2-T24 | 0.958985 | -369 | TCGAAACCGAAACT | | - |
| USP18 | 0.939209 | -85 | AGGAAAGCGAAACT | | - |
| GBP2 | 0.93129 | -504 | CTGAAAGTGAAAGT | | - |
| IFIT3 | 0.930494 | -158 | AGGAAAGTGAAACT | | - |
| IFIT3B | 0.930494 | -190 | AGGAAAGTGAAACT | | - |
| MX2 | 0.929133 | -941 | ATGAAAGTGAAACT | | + |
| IFI47 | 0.925265 | -36 | ACAAAAGTGAAACT | | - |
| SLFN4 | 0.925265 | -227 | ACAAAAGTGAAACT | | - |
| PARP10 | 0.925265 | 0 | ACAAAAGTGAAACT | | - |
| TNFSF10 | 0.922431 | -55 | CCAAAAGTGAAACT | | - |
| UBA7 | 0.922431 | -29 | CCAAAAGTGAAACT | | - |
| RSAD2 | 0.919583 | -122 | CTGAAAATGAAAGT | | - |
| TRIM34A | 0.919033 | 8 | AAGAAACTGAAACT | | - |
| CXCL10 | 0.917424 | -215 | TGGAAAGTGAAACT | | + |
| IFIT1BL2 | 0.917424 | -53 | TGGAAAGTGAAACT | | + |
| IFIT1 | 0.917424 | -106 | TGGAAAGTGAAACT | | - |
| ISG20 | 0.916198 | -311 | CAGAAACTGAAACT | | + |
| SERPINB9 | 0.915448 | -48 | GAGAAAGTGAAAGT | | - |
| IRGM1 | 0.914678 | -100 | TAGAAACCGAAACT | | + |
| TRIM21 | 0.912534 | 11 | AGGAAACTGAAAGT | | + |
| OAS3 | 0.912456 | -77 | GAGAAAACGAAAGT | | + |
| PARP14 | 0.911278 | -56 | ATGAAAGCGAAAGA | | + |
| TRIM56 | 0.907306 | -6 | ACAAAACTGAAAGT | | - |
| XAF1 | 0.907306 | -19 | ACAAAACTGAAAGT | | - |
| GBP3 | 0.905963 | -55 | TAGAAACTGAAACT | | - |
| CD274 | 0.903844 | -340 | ACGAAACTAAAAGT | | - |
| GBP7 | 0.903346 | -55 | CTGAAACTGAAACT | | - |
| RTP4 | 0.903187 | -76 | TGGAAACCGAAACT | | + |
| ISG15 | 0.902317 | -68 | CCGAAACAGAAAAT | | + |
| ZBP1 | 0.900109 | -96 | TCGAAATCGAAATT | | - |
| OAS1B | 0.899464 | -21 | TGGAAACTGAAAGT | | + |
| APOL9A | 0.899464 | -61 | TGGAAACTGAAAGT | | - |
| APOL9B | 0.899464 | -88 | TGGAAACTGAAAGT | | - |
| SLFN8 | 0.895973 | -138 | GGGAAAACGAAACT | | - |
| IFIT1BL1 | 0.895037 | -106 | CAGAAAATGAAACC | | - |
| TOR3A | 0.887225 | -470 | AGGAAAATGAAACA | | - |
| IFI205 | 0.883058 | 21 | GAGAAAGTGAAAAA | | - |
| GBP5 | 0.88277 | -161 | TCTAAACTGAAATT | | - |
| CD40 | 0.879632 | -502 | AAGAAAGAGAAACT | | + |
| DDX58 | 0.879224 | 0 | AGGAAATCGAAACT | | - |
| OAS2 | 0.879224 | -78 | AGGAAATCGAAACT | | - |
| TAP1 | 0.878906 | -563 | GTGAAAGCGAAAGC | | + |
| GM12250 | 0.878768 | -48 | ATGAAACTGAAAGC | | - |
| IGTP | 0.875933 | -36 | CTGAAACTGAAAGC | | + |
| IRGM2 | 0.875933 | -66 | CTGAAACTGAAAGC | | + |
| NOS2 | 0.875728 | -885 | ATGAAAGTGAAATA | | - |
| DHX58 | 0.874619 | -6 | ATGAAACTGAAACA | | - |
| SP100 | 0.871467 | -49 | AGAAAAACGAAAGT | | - |
| IRG1 | 0.870451 | -67 | ACAAAAGTGAAAGG | | + |
| DTX3L | 0.866657 | -845 | CCAAAAGCAAAACT | | + |
| EIF2AK2 | 0.864411 | 16 | GGGAAAACGAAACA | | + |
| EPSTI1 | 0.863175 | -314 | TGGAAAGTGAAATC | | - |
| OASL1 | 0.862913 | -159 | ACAAAAGAGAAACT | | + |
| SP110 | 0.862752 | 7 | AGAAAAATGAAAGT | | - |
| RILPL1 | 0.857785 | -772 | ACAAAAACAAAACT | | + |
| CCL22 | 0.857333 | -897 | CAGAAAGTGGAAAT | | + |
| IFIH1 | 0.855512 | 30 | TCGAAACAGAAACC | | + |
| TRIM14 | 0.855474 | -4 | CAGAAATCGAAACC | | - |
| ADAR | 0.855095 | -808 | CTGAAAATAAAAGT | | + |
| TARM1 | 0.855071 | -341 | TGGAAAGAGAAACT | | + |
| CCL5 | 0.853544 | -132 | CATAAAATGAAAAC | | - |
| AI607873 | 0.851859 | -43 | GGGAAATCGAAAAT | | + |
| GM5431 | 0.850228 | -399 | CAGAAAGAGAAAGA | | + |
| IFI44 | 0.84368 | -8 | CGAAAACTGAAACT | | - |
| IL15 | 0.842898 | -306 | AGAAAAGTGAAAGA | | - |
| MS4A6C | 0.841786 | -135 | AAGAAAGTGAACTT | | + |
| MNDAL | 0.840527 | -568 | AAGAAAAAGAAAAA | | - |
| GM6548 | 0.840527 | -571 | AAGAAAAAGAAAAA | | + |
| IFIT2 | 0.83864 | -640 | GGGAAAGTAAAAAT | | - |
| SLFN5 | 0.837806 | -163 | TTCAAAGTGAAAAA | | + |
| CFB | 0.836877 | -935 | CCCAAAACAAAAAC | | + |
| CCL2 | 0.836435 | -113 | TGGAAAGTGGAAGT | | - |
| TRIM30A | 0.836396 | -38 | GAGAAAAAGAAACT | | - |
| TRIM30D | 0.836396 | -43 | GAGAAAAAGAAACT | | - |
| PHF11B | 0.836396 | -52 | GAGAAAAAGAAACT | | + |
| PHF11A | 0.836396 | -31 | GAGAAAAAGAAACT | | + |
| SAMD9L | 0.836047 | 29 | TCGAAACTGGAAAC | | + |
| SRC | 0.834719 | -562 | TTGAAAGTGAATTT | | + |
| PYHIN1 | 0.834716 | -572 | GCTAAAGCCAAAAT | | + |
| PHF11D | 0.83334 | -7 | CGGGAAACGAAACT | | + |
| PARP11 | 0.830569 | -614 | AAGAAAGCAAAAAG | | - |
| OASL2 | 0.825379 | -247 | ACAAAAACAAAACC | | + |
| IL6 | 0.824672 | -288 | AGAAAACTGAAATT | | - |
| PPM1K | 0.824065 | -22 | CGGAAAATAAAAAA | | - |
| BCL2A1D | 0.824042 | -56 | GTGAAAGCCAAAGT | | - |
| ZUFSP | 0.823479 | -101 | GTGCAAGCGAAAGT | | + |
| HERC6 | 0.822947 | -506 | TAGAAAGCCAAAGC | | - |
| CLEC2D | 0.818711 | -180 | ACGAAACACAAAGT | | + |
| MX1 | 0.817876 | -135 | TCAGAAACGAAACT | | - |
| CD69 | 0.817776 | -637 | AGGAAACAGAAAGC | | - |
| PARP9 | 0.816931 | -250 | ACCAAAACCAAAAC | | + |
| TRIM25 | 0.816272 | -12 | TCGAAACTGAACAG | | - |
| MTMR7 | 0.814812 | -670 | GTGAAAGTGAGAGT | | - |
| SLC7A2 | 0.813374 | -499 | ATGAAATCAAAACT | | - |
| LGALS9 | 0.812784 | 13 | AGGAAACAGAAACC | | - |
| CCL12 | 0.812674 | -187 | TAGACAGCGAAACA | | - |
| IFI204 | 0.811567 | -163 | GGGAAATTGAAAGC | | + |
| MNDA | 0.811567 | -39 | GGGAAATTGAAAGC | | + |
| TRIM12A | 0.811422 | 8 | ATGAAACAGAAACC | | - |
| TRIM12C | 0.811422 | -22 | ATGAAACAGAAACC | | - |
| SP140 | 0.809217 | -279 | TCAAAAATGATACT | | + |
| TRAFD1 | 0.808399 | -918 | ACAAAACAGAAACA | | + |
| CCL7 | 0.806525 | -903 | TGGAAAACGACAAG | | + |
| IL1A | 0.803145 | -870 | TGGGAACTGAAACT | | + |

**Supporting Information Table 2:** List of primers used in qRT-PCR studies

| **Gene symbol** | **Forward Sequence (5' -> 3')** | **Reverse Sequence (5' -> 3')** |
| --- | --- | --- |
| ***CCL2*** | TAA AAA CCT GGA TCG GAA CCA AA | GCA TTA GCT TCA GAT TTA CGG GT |
| ***CCL7*** | CCACATGCTGCTATGTCAAGA | ACACCGACTACTGGTGATCCT |
| ***CCL9*** | CCCTCTCCTTCCTCATTCTTACA | AGTCTTGAAAGCCCATGTGAAA |
| ***CXCL10*** | TGC TGG GTC TGA GTG GGA CT | CCC TAT GGC CCT CAT TCT CAC |
| ***IL1RN*** | GCTCATTGCTGGGTACTTACAA | CCAGACTTGGCACAAGACAGG |
| ***IRG1*** | GGCACAGAAGTGTTCCATAAAGT | GAGGCAGGGCTTCCGATAG |
| ***IRF7*** | GCGTACCCTGGAAGCATTTC | GCACAGCGGAAGTTGGTCT |
| ***IL6*** | TAG TCC TTC CTA CCC CAA TTT CC | TTG GTC CTT AGC CAC TCC TTC |
| ***GAPDH*** | TGCGACTTCAACAGCAACTC | CTTGCTCAGTGTCCTTGCTG |
